# A novel engineered IL-21 receptor arms T-cell receptor-engineered T cells (TCR-T cells) against hepatocellular carcinoma

**DOI:** 10.1038/s41392-024-01792-6

**Published:** 2024-04-20

**Authors:** Wei Zhu, Zhiming Zhang, Jinzhang Chen, Xiaolan Chen, Lei Huang, Xiaoyong Zhang, Xuan Huang, Na Ma, Weikang Xu, Xuan Yi, Xinyu Lu, Xin Fu, Siwei Li, Guoheng Mo, Yiyue Wang, Guosheng Yuan, Mengya Zang, Qi Li, Xiaotao Jiang, Yajing He, Sha Wu, Yukai He, Yongyin Li, Jinlin Hou

**Affiliations:** 1 State Key Laboratory of Organ Failure Research, Guangdong Provincial Key Laboratory of Viral Hepatitis Research, Department of Infectious Diseases, Nanfang Hospital, Southern Medical University, Guangzhou, China; 2 Institute of Cellular Medicine, Newcastle University Medical School, Newcastle, UK; 3 Department of Pathology, The First People’s Hospital of Foshan, Foshan, China; 4 Dermatology Hospital, Southern Medical University, Guangzhou, China; 5 Department of Immunology, School of Basic Medical Sciences, Southern Medical University, Guangzhou, China; 6 Microbiome Medicine Center, Department of Laboratory Medicine, Zhujiang Hospital, Southern Medical University, Guangzhou, China; 7 Key Laboratory of Proteomics of Guangdong Province, Demonstration Center for Experimental Education of Basic Medical Sciences of China, Guangzhou, China; 8 Medical College of Georgia, Augusta University, 1120 15th Street, Augusta, GA USA

**Keywords:** Immunotherapy, Drug development

## Abstract

Strategies to improve T cell therapy efficacy in solid tumors such as hepatocellular carcinoma (HCC) are urgently needed. The common cytokine receptor γ chain (γ_c_) family cytokines such as IL-2, IL-7, IL-15 and IL-21 play fundamental roles in T cell development, differentiation and effector phases. This study aims to determine the combination effects of IL-21 in T cell therapy against HCC and investigate optimized strategies to utilize the effect of IL-21 signal in T cell therapy. The antitumor function of AFP-specific T cell receptor-engineered T cells (TCR-T) was augmented by exogenous IL-21 in vitro and in vivo. IL-21 enhanced proliferation capacity, promoted memory differentiation, downregulated PD-1 expression and alleviated apoptosis in TCR-T after activation. A novel engineered IL-21 receptor was established, and TCR-T armed with the novel engineered IL-21 receptors (IL-21R-TCR-T) showed upregulated phosphorylated STAT3 expression without exogenous IL-21 ligand. IL-21R-TCR-T showed better proliferation upon activation and superior antitumor function in vitro and in vivo. IL-21R-TCR-T exhibited a less differentiated, exhausted and apoptotic phenotype than conventional TCR-T upon repetitive tumor antigen stimulation. The novel IL-21 receptor in our study programs powerful TCR-T and can avoid side effects induced by IL-21 systemic utilization. The novel IL-21 receptor creates new opportunities for next-generation TCR-T against HCC.

## Introduction

Primary liver cancer is the sixth most commonly diagnosed cancer and the third leading cause of cancer death with approximately 905,677 new cases and 830,180 deaths worldwide in 2020.^1^ Hepatocellular carcinoma (HCC) comprises a large proportion (75–85% of cases) of primary liver cancer. Current therapies for early-stage HCC patients include local ablation, resection and liver transplantation, yielding over 50% 5-year overall survival rates post-treatment.^2^ However, therapies for advanced-stage HCC patients are limited, and the median overall survival durations are 10.7-14.6 months in advanced-stage HCC patients who received recommended systemic therapy such as Sorafenib or Lenvatinib.^3,4^ Thus, novel therapeutic strategies to extend the overall survival of advanced-stage HCC patients are urgently needed. Adoptive T cell therapy is a form of immunotherapy that using effector cells sensitized or modified ex vivo^5^ to eliminate the tumor cells in cancer patients. Current targets for adoptive T cell therapy in HCC patients include tumor-associated antigens, cancer-testis antigens and viral-derived antigens with numerous ongoing related clinical trials.^6,7^ However, many obstacles limiting the efficacy of adoptive T cell therapy in HCC patients that still need to be overcome, such as the poor access of transferred T cells to the tumor due to vasculature restriction or physical barrier of the stroma,^8^ the dysfunction of tumor-infiltrating T cells caused by the tumor microenvironment that composed of immunosuppressive cells and cytokines,^9^ and the poor in vivo expansion and persistence of transferred T cells.^10^ Enhancement of in vivo expansion and persistence of transferred T cells^10,11^ emerges as a promising strategy to augment the clinical efficacy of adoptive T-cell therapy in patients with solid tumors.

The common cytokine receptor γ chain (γ_c_) family, including IL-2, IL-4, IL-7, IL-9, IL-15, and IL-21, plays fundamental roles in T cell development and differentiation.^12^ IL-2 have received FDA approval for cancer treatment due to the ability to support the proliferation and function of adoptively transferred T cells.^13^ IL-7 and IL-15 have been used to improve the antitumor activity of adoptive T cell therapy^14,15^ due to their capacity of memory maintenance in CD8^+^ T cells.^16^ As a member of γ_c_ family cytokines, IL-21 is primarily produced by Th17 and Tfh subsets within activated CD4^+^ T cells.^17^ Notably, IL-21 has been demonstrated to prevent T cell differentiation and preserve a naive-like phenotype of T cells^18,19^ during the ex vivo expansion. This property has led researchers to utilize IL-21 to generate “younger” T cells, characterized by superior proliferation potential and enhanced antitumor capacity.^20,21^ Meanwhile, IL-21 also supports the effector function of cytotoxic CD8^+^ T cells by activating the Basic Leucine Zipper ATF-Like Transcription Factor pathway.^21,22^ Within the tumor microenvironment, IL-21 is secreted by tumor-specific Tfh cells to bolster the effector function of tumor-infiltrating CD8^+^ T cells^23^ and is regarded as a pivotal cytokine in the CD4^+^ T and CD8^+^ T cell interaction. Thus, using the biological effect of IL-21 emerges as a promising strategy to augment the efficacy of adoptive T-cell therapy in HCC patients.

Despite the promise of cytokine-based therapies, numerous hurdles persist and need to be overcome, including the pharmacokinetic challenges and side effects. The short half-life of cytokines limits in vivo efficacy and necessitates frequent dosing. IL-21, a pleiotropic cytokine exerting influence on various immune cell types including B cells, NK cells, and macrophages,^24^ faces challenges in its systemic administration due to dose-limiting toxicity observed in high-dose regimens during a Phase II clinical trial for IL-21 monotherapy^25^ in metastatic melanoma patients. Alternative approaches such as establishing IL-21 fusion protein to prolong the half-life or engineering T cells to secret cytokines^26^ still carry a risk of toxicities by the cytokine accumulation. Hence, the quest for novel strategies ensuring the safe and effective utilization of IL-21 in adoptive T cell therapy remains imperative.

In this study, we evaluated the combined antitumor effects of AFP-specific TCR-T (AFP-TCR-T) with γ_c_ family cytokines including IL-7, IL-15 and IL-21 respectively in a repetitive coculture assay and selected IL-21 as our objective to promote antitumor effects of AFP-TCR-T. The biological effects of exogenous IL-21 to AFP-TCR-T were further investigated during the in vitro expanding phase and in the xenograft model. After determining the superior antitumor function of IL-21-supplemented TCR-T, we established a mutant IL-21 receptor that transmitted constitutive IL-21 signal without the external IL-21. TCR-T with the mutant IL-21 receptor (IL-21R-TCR-T) showed augmented proliferation capacity and killing function against high-load tumor antigens compared with conventional TCR-T in coculture assay and xenograft model. Phenotypic analysis showed that IL-21R-TCR-T retained a less differentiated, exhausted and apoptotic phenotype than conventional TCR-T upon repetitive tumor antigen stimulation. These results establish a novel strategy providing the IL-21 signal to the engineered T cells and preventing non-specific bystander T-cell activation associated with transgenic cytokine expression. This innovative approach holds promise for application in engineered T cell therapy against hepatocellular carcinoma and potentially other solid tumors.

## Results

### γ_c_ family cytokine IL-21 was identified as a promising target to improve adoptive T-cell therapy efficacy against HCC

**Fig. 1:**
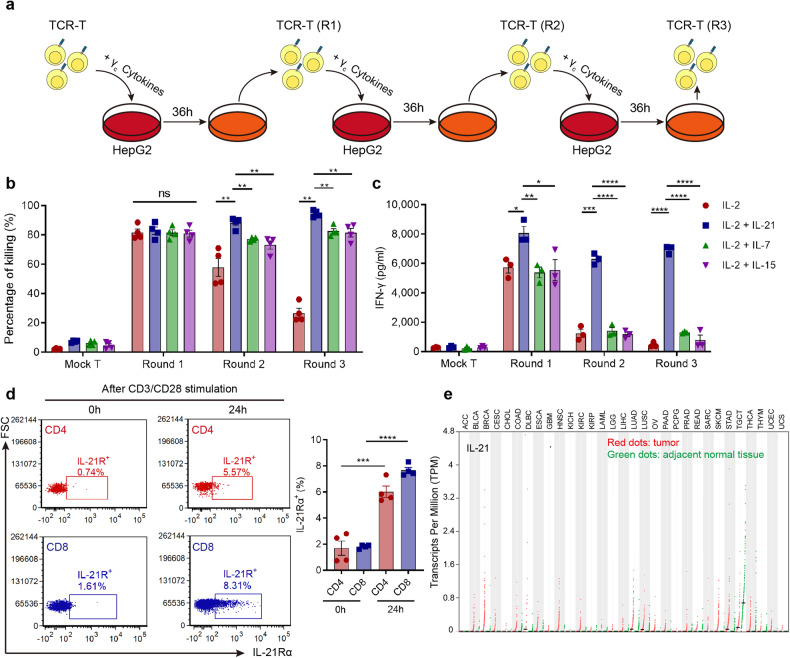
Identification of the γ_c_ family cytokine IL-21 as a potential target to improve adoptive T cell therapy efficacy against HCC. **a** The schematic of the repetitive coculture assay. After the first round of 36 h coculture with HepG2, TCR-T were rechallenged with fresh HepG2 for another 36 h. Then the TCR-T were rechallenged with another fresh HepG2 for 36 h again if applicable. **b** The CTL activities of mock-transduced T cells or TCR-T after 36 h coculture with HepG2 in the presence of different cytokines were measured by LDH assay (*n* = 4). **c** The supernatant IFN-γ levels after 36 h coculture of mock-transduced T or TCR-T cells with HepG2 in the presence of different cytokine combinations were measured by ELISA (*n* = 3). **d** The expression of the IL-21Rα chain in human T cells before and after 24 h anti-CD3/CD28 activation was detected by flow cytometry. The percentage of IL-21Rα positive cells before and after 24 h anti-CD3/CD28 activation in CD4^+^ and CD8^+^ T cells was shown (*n* = 4). **e** Expression (transcripts per million) of IL-21 in tumor (red dots) and adjacent normal tissue (green dots) from different patients with cancer plotted using TCGA data. Data were shown as mean ± SEM, *P < 0.05, **P < 0.01, ***P < 0.001, ****P < 0.0001, NS not significant

γ_c_ family cytokines IL-7, IL-15 or IL-21 were supplemented during the effector phase of AFP-TCR-T in the presence of IL-2 using a repetitive coculture assay with HLA-A2^+^ HepG2 cell lines (Fig. 1a). AFP-TCR-T supplemented with IL-7, IL-15 or IL-21 showed enhanced killing activity against HepG2 under the microscope compared with only IL-2 (Supplementary Fig. 1a), and the augmented killing activity was most evident in AFP-TCR-T supplemented with IL-21. Mock-transduced T cells with IL-7, IL-15 and IL-21 showed poor killing activity against HepG2 (Supplementary Fig. 1a), indicating that the augmented killing activity relied on the specific recognition of AFP. The quantified killing rate (Fig. 1b) showed that both IL-7, IL-15 and IL-21 enhanced the sustained killing capacity of AFP-TCR-T and IL-21-supplemented AFP-TCR-T showed the highest killing rate among all the groups, especially after the two and three round coculture. Meanwhile, IL-21-supplemented AFP-TCR-T showed a remarkable increase in IFN-γ secretion compared with other groups (Fig. 1c), suggesting that IL-21-supplemented TCR-T sustained a more robust effector function than IL-7 and IL-15 in the repetitive coculture assay. Due to the distinguished improvement of TCR-T effector function, we next evaluated the feasibility of IL-21 application in adoptive T-cell therapy. We found that the expression of the IL-21 receptor was significantly elevated in human CD4^+^ and CD8^+^ T cells after 24 h CD3/CD28 activation (Fig. 1d), which implied that the IL-21 signal is required in activated T cells. Meanwhile, Analysis of RNA-seq data from the Cancer Genome Atlas showed that IL-21 transcript was barely detectable in numerous tumors including HCC (Fig. 1e), predicting an absence of IL-21 in the tumor microenvironment. Therefore, supplementing the IL-21 signal to AFP-TCR-T may support its antitumor function within the HCC tumor microenvironment, which is deprived of the IL-21 signal.

### IL-21 promoted the proliferation and in vivo anti-HCC capacity of AFP-TCR-T

**Fig. 2:**
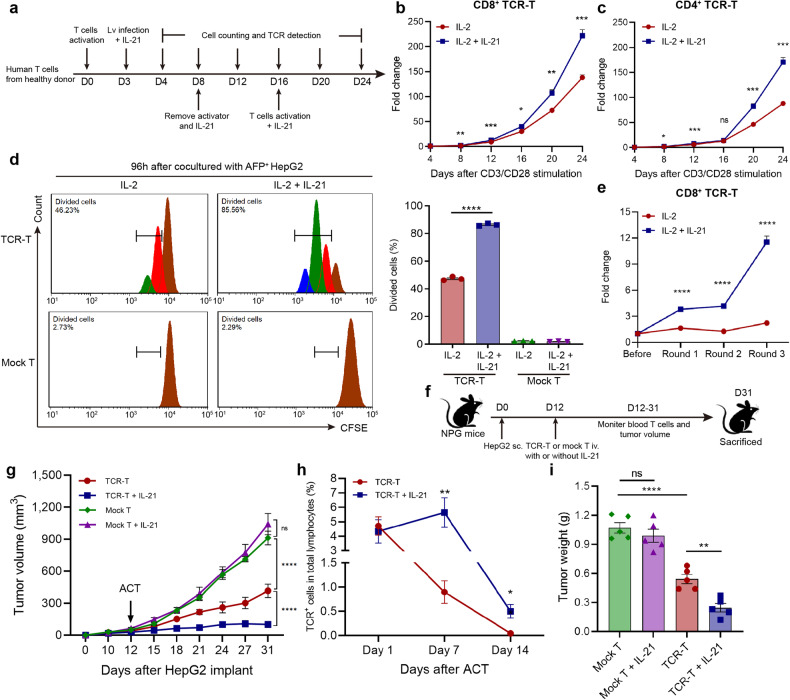
IL-21 promoted activation-induced proliferation and improved in vivo anti-HCC function of TCR-T. **a** The process of generating human TCR-T and IL-21 stimulation was shown. The cell growth of TCR-T during expanding was monitored. Mock transduced T cells had undergone the same expanding procedure without lv transduction. **b**, **c** The proliferating fold change of CD8^+^ and CD4^+^ TCR-T after CD3/CD28 activation in the presence of IL-2 (10 ng/ml) or IL-2 (10 ng/ml) and IL-21 (20 ng/ml) was shown respectively (*n* = 3). **d** TCR-T were labeled by CFSE, cultured with HepG2 for 96 h in the presence of IL-2 or IL-2 and IL-21 and analyzed by flow cytometry. The percentage of divided cells in the CD8^+^ subset was shown (*n* = 3). The brown peak indicated the first proliferating generation, the red indicated the second, the green indicated the third and the blue indicated the fourth. **e** The fold change of CD8^+^ TCR-T percentage in total T cells during the repetitive coculture assay was shown (*n* = 3). **f** Schematics of subcutaneous HepG2 model establishment and adoptive transfer process in NPG mice. **g** The mice tumor volume in each group after HepG2 implantation and mock-transduced T or TCR-T cell transfer was shown (*n* = 5). **h** The TCR-T percentage in total lymphocytes (mice and human lymphocytes) in mice peripheral blood 1, 7 and 14 days after TCR-T transfer was shown (*n* = 5). **i** Tumors were isolated at the end of the experiment from tumor-bearing mice receiving mock-transduced T or TCR-T treatment with or without IL-21 and the weight of isolated tumors was shown (*n* = 5). Data were shown as mean ± SEM, *P < 0.05, **P < 0.01, ***P < 0.001, ****P < 0.0001, NS not significant, ACT adoptive cell transfer

To further investigate the efficacy of IL-21 in adoptive T-cell therapy, human AFP-TCR-T was supplemented with exogenous IL-21 in the presence of IL-2 during expanding phase (Fig. 2a). We found that the cell growth of TCR-T after CD3/CD28 activation was boosted by IL-21 both in CD8^+^ and CD4^+^ population as the fold change of cell counting in IL-21-supplemented TCR-T was significantly higher than TCR-T without IL-21 supplement after 24 days expansion (Fig. 2b, c). The proliferation of AFP-TCR-T after HepG2 tumor stimulation was further monitored. We found that the divided cell proportion in CD8^+^ TCR-T with IL-21 was significantly higher than TCR-T without IL-21 after 96 h coculture with HepG2 (Fig. 2d), demonstrating the superior proliferation capacity of IL-21-supplemented TCR-T. Mock-transduced T cells with IL-21 showed poor proliferation after HepG2 stimulation, indicating that the proliferation augmented by IL-21 depends on the activation of T cells by specific antigens. Meanwhile, the CD8^+^ TCR-T percentage showed more increase in IL-21-supplemented TCR-T after three rounds of HepG2 stimulation (Fig. 2e). These results indicated that IL-21 promoted the proliferation of AFP-TCR-T during tumor antigen stimulation.

We next investigated whether IL-21 can enhance the in vivo antitumor efficacy of AFP-TCR-T. Immunocompromised NPG mice were subcutaneously implanted with HepG2 and received mock-transduced T cells or AFP-TCR-T intravenously with or without IL-21 (Fig. 2f). We found that tumor growth was significantly inhibited by AFP-TCR-T transferring, while AFP-TCR-T transferred with IL-21 showed more powerful antitumor capacity than without IL-21 (Fig. 2g). Mice were sacrificed at the end of the experiment and the tumors were isolated and weighed. The volume and weight (Fig. 2i and Supplementary Fig. 1b) of tumors in mice receiving AFP-TCR-T treatment were significantly lower than mock-transduced T cells and were lowest in mice receiving AFP-TCR-T with IL-21, indicating that IL-21 augmented the in vivo antitumor function of AFP-TCR-T. Meanwhile, the percentage of AFP-TCR-T in peripheral blood was significantly higher in mice with IL-21 supplement at day 7 and day 14 after TCR-T transferred (Fig. 2h), demonstrating that IL-21 improved the in vivo persistence of AFP-TCR-T. The immunohistochemistry of mouse TCRβ (to define AFP-TCR^+^ cells) in tumor 7 days after transferring showed enhanced tumor-infiltrating TCR-T cells in mice receiving AFP-TCR-T with IL-21 (Supplementary Fig. 1c), demonstrating that IL-21 increased the amounts of tumor-infiltrating TCR-T cells, which led to the augmented in vivo antitumor function of AFP-TCR-T.

### IL-21 promoted TCR-T stemness and alleviated exhaustion and apoptosis in AFP-TCR-T

**Fig. 3:**
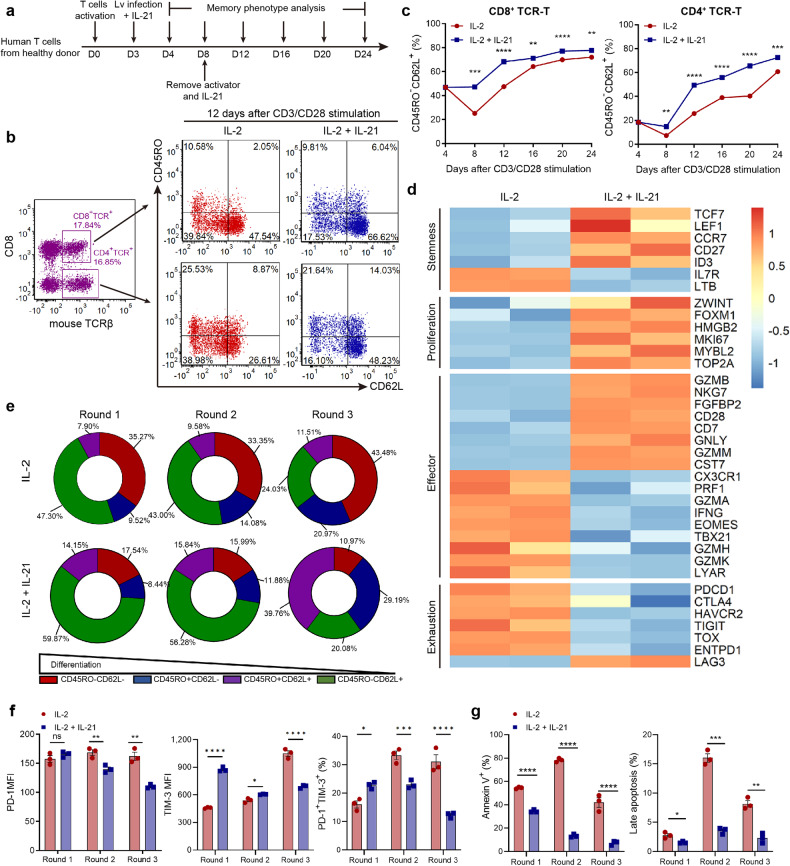
IL-21 promoted memory differentiation of TCR-T after CD3/CD28 and tumor stimulation. **a** The memory phenotype of CD8^+^ and CD4^+^ TCR-T was monitored during the TCR-T generation process. **b** The CD45RO and CD62L expression of TCR-T detected by flow cytometry after 12 days of anti-CD3/CD28 activation in the presence of IL-2 (10 ng/ml) or IL-2 (10 ng/ml) + IL-21 (20 ng/ml) was shown. Each figure shown was representative of three replicates. **c** The percentage of CD45RO^-^CD62L^+^ population in CD8^+^ and CD4^+^ TCR-T from Day 4 to Day 24 after anti-CD3/CD28 activation in the presence of IL-2 or IL-2 + IL-21 was shown respectively (*n* = 3). **d** The heatmap of stemness, proliferation, effector and exhaustion-related gene expression in IL-2 or IL-2 + IL-21 maintained T cells during CD3/CD28 induced expansion. **e** The CD45RO and CD62L expression of CD8^+^ TCR-T after 36 h coculture with HepG2 in the presence of IL-2 or IL-2 + IL-21 was detected by flow cytometry. The percentage of different subsets determined by CD45RO and CD62L in CD8^+^ TCR-T after 36 h coculture with HepG2 in the presence of IL-2 or IL-2 + IL-21 was shown. **f** The PD-1 and TIM-3 expression of CD8^+^ TCR-T after cocultured with HepG2 in the presence of IL-2 or IL-2 + IL-21 was measured by flow cytometry. The percentage of PD-1^+^TIM-3^+^ proportion and the MFI of PD-1 and TIM-3 were shown (*n* = 3). **g** The viability of CD8^+^ TCR-T after 36 h coculture with HepG2 was measured by flow cytometry. The percentage of Annexin V positive and late apoptosis cells (7-AAD^+^) was shown (*n* = 3). Data were shown as mean ± SEM, *P < 0.05, **P < 0.01, ***P < 0.001, ****P < 0.0001, NS not significant

After determining the superior proliferation capacity and antitumor function, we investigated the detailed phenotypic alteration in IL-21-supplemented TCR-T. IL-21 has been reported to maintain a naive-like phenotype in T cells and promote the differentiation of stem-cell memory T cell population with superior proliferation potential and long-term antitumor capacity.^10^ Therefore, the memory phenotype of IL-21-supplemented TCR-T was monitored during the ex vivo expanding phase after CD3/CD28 activation (Fig. 3a). We found that IL-21-supplemented TCR-T showed a higher percentage of CD45RO^-^CD62L^+^ naive-like population than conventional IL-2-maintained TCR-T after CD3/CD28 activation both in the CD4^+^ and CD8^+^ subset (Fig. 3b, c). The increased CD45RO^-^CD62L^+^ naive-like population in IL-21-supplemented TCR-T persists in the ex vivo expansion process from day 4 to day 24 (Fig. 3c and Supplementary Fig. 10a–c). The RNA-seq data showed that stemness-related genes such as *TCF7, LEF1* and *CCR7* were upregulated, effector-related genes such as *EOMES, TBX21, and PRF1* and exhaustion-related genes such as *PDCD1*, *CTLA4*, *TIGIT* and *TOX* were downregulated in IL-21-supplemented TCR-T compared with conventional IL-2-maintained TCR-T (Fig. 3d) during expansion. The phenotypic change of IL-2 or IL-21-supplemented TCR-T during repetitive tumor antigen stimulation was also monitored. We found that IL-21-supplemented TCR-T showed a higher proportion of central-memory T cell (CD45RO^+^CD62L^+^) and naive-like T cell (CD45RO^-^CD62L^+^) subsets during repetitive coculture with HepG2 (Fig. 3e and Supplementary Fig. 2a, b). These results demonstrated that IL-21 maintained stemness and reduced terminal effector differentiation in AFP-TCR-T after activation. Considering the superior effector function of IL-21-supplemented TCR-T in the tumor coculture assay, the IL-21 signal may help to generate a unique “long-live effector” phenotype of TCR-T.

We then characterized the exhaustion and apoptosis of TCR-T in the repetitive coculture assay. We found that the exhaustion related markers PD-1 and TIM-3 expression and percentage of PD-1^+^TIM-3^+^ subsets was lower in CD8^+^ IL-21-supplemented AFP-TCR-T than IL-2-supplemented after the three rounds coculture assay with HepG2 (Fig. 3f and Supplementary Fig. 2d). Meanwhile, the percentage of Annexin V^+^ or 7-AAD^+^ population was lower in IL-21-supplemented AFP-TCR-T during repetitive coculture with HepG2 (Fig. 3g and Supplementary Fig. 2c). These results indicated that IL-21 alleviated the exhaustion and promoted the survival of AFP-TCR-T during persistent antigen stimulation.

### Establishment of IL-21R-TCR-T and constitutive IL-21 signal verification

**Fig. 4:**
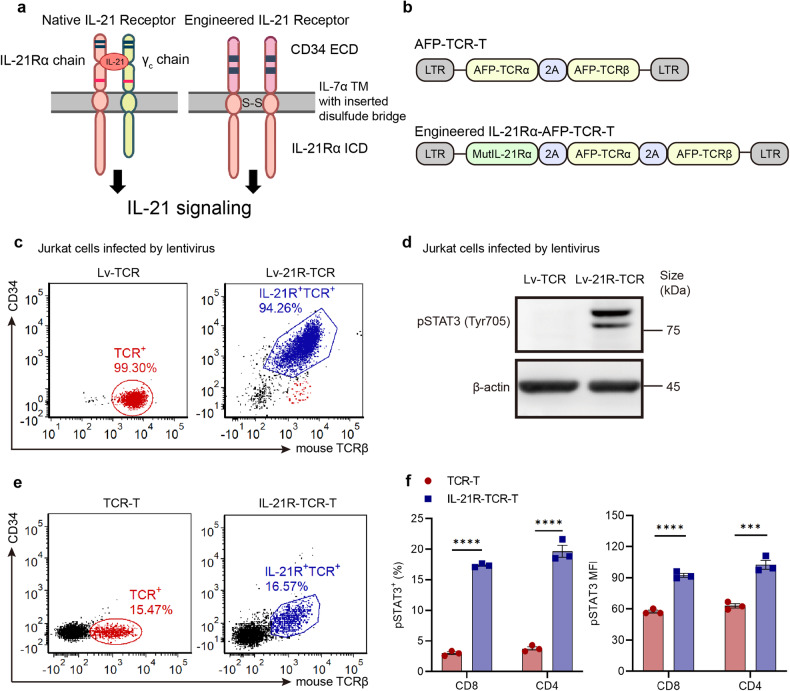
Design and establishment of IL-21R-TCR-T. **a** Schematic of the native IL-21 receptor and engineered IL-21R receptor. **b** Schematics of the lentivirus vectors of AFP-TCR-T and IL-21R-AFP-TCR-T. **c** The expression of TCR and CD34 in lentivirus-infected Jurkat cells was measured by flow cytometry. **d** The STAT3 phosphorylation in lentivirus-infected Jurkat cells was quantified by western blot analysis. **e** The AFP-TCR and CD34 expression on human TCR-T, IL-21R-TCR-T was measured by flow cytometry. **f** STAT3 phosphorylation levels in human TCR-T and IL-21R-TCR-T were measured by flow cytometry. The percentage of pSTAT3^+^ cells and MFI of pSTAT3 in CD8^+^ and CD4^+^ TCR-T was shown (*n* = 3). Data were shown as mean ± SEM, *P < 0.05, **P < 0.01, ***P < 0.001, ****P < 0.0001, NS not significant, ECD extracellular domain, TM transmembrane domain, ICD intracellular domain, LTR long terminal repeats, pSTAT3 phosphorylated STAT3

After determining the biological effect of the IL-21 signal in TCR-T, we next designed and established a novel IL-21 receptor transmitting constitutive phosphorylated STAT3 signal independent of external ligand. Two IL-21Rα chains were linked by a disulfide bond in the transmembrane domain to create an IL-21Rα homodimer. The extracellular domain of the IL-21Rα chain homodimer was replaced by ectodomains derived from CD34 to provide a means of detecting transduced cells (Fig. 4a). The engineered IL-21Rα homodimer was then linked to TCRα and β chain in a lentivirus vector (Fig. 4b). After lentiviral transduced, the engineered IL-21R and AFP-specific TCR were co-expressed in Jurkat cells (Fig. 4c) or human T cells (Fig. 4e). AFP-specific TCR was also co-expressed with CD34 ectodomain to generate AFP-TCR-T with CD34 ectodomain (CD34-TCR-T) to exclude the potential effects of CD34 expression (Supplementary Fig. 3a). As expected, engineered IL-21R expression led to constitutive STAT3 phosphorylation in Jurkat cells (Fig. 4d). Meanwhile, human TCR-T with engineered IL-21R expression (IL-21R-TCR-T) also showed higher STAT3 phosphorylation than conventional TCR-T and CD34-TCR-T in the absence of external IL-21, confirming the constitutive IL-21 signal (weaker than exogenous IL-21 stimulation) in IL-21R-TCR-T (Fig. 4f and Supplementary Fig. 3b, c). To investigate the persistence of the STAT3 signaling activated by the engineered IL-21R, the levels of STAT3 phosphorylation were monitored in IL-21R-TCR-T after 30, 45 and 60 days in vitro culture, respectively (Supplementary Fig. 3f, g). The results showed IL-21R-TCR-T maintained a consistent level of STAT3 phosphorylation and higher than conventional TCR-T after 60 days in vitro culture, indicating that the STAT3 signal transmitted by the engineered IL-21R was stable.

### IL-21R-TCR-T showed augmented activation-induced proliferation and superior antitumor function

**Fig. 5:**
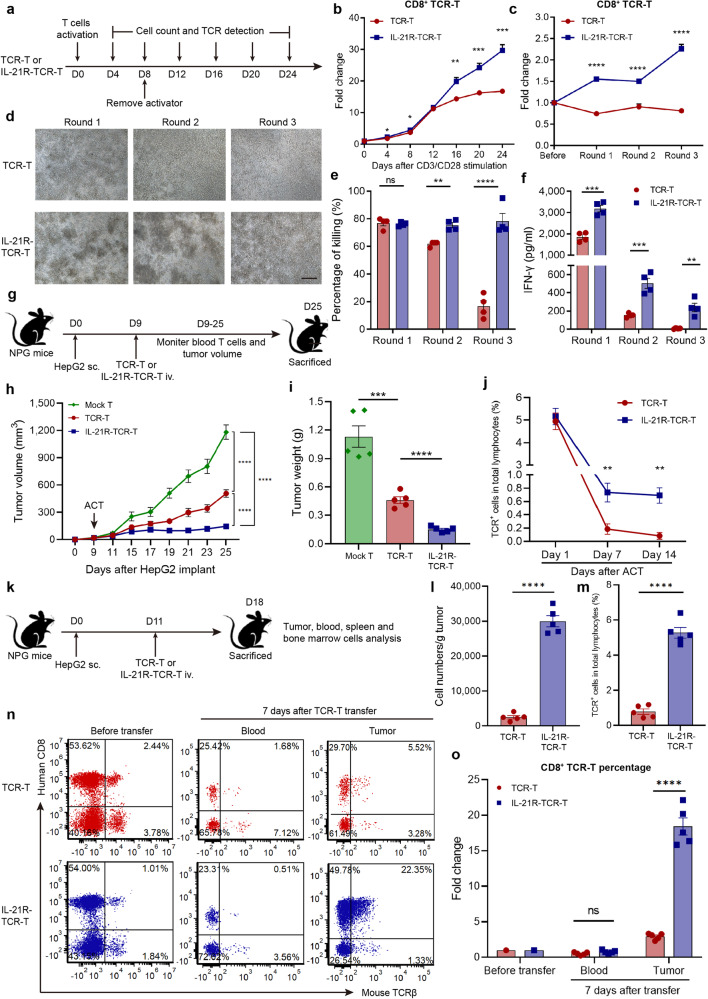
Superior proliferation and anti-HCC capacity of IL-21R-TCR-T. **a** The process of TCR-T and IL-21R-TCR-T receiving CD3/CD28 activation was shown. The cell number and TCR^+^ subset percentage were monitored every 4 days during the process. **b** The proliferating fold change of CD8^+^ TCR-T and IL-21R-TCR-T after CD3/CD28 activation was shown (*n* = 3). **c** The percentage of CD8^+^ TCR^+^ proportion in TCR-T and IL-21R-TCR-T during three rounds repeated coculture with HepG2 was shown (*n* = 3). **d** Representative pictures of HepG2 after 36 h coculture (Round 1, Round 2 and Round 3, the same procedure as Fig. 1a) with conventional TCR-T or IL-21R-TCR-T were shown. Scale bar = 100 µm. **e** The CTL activity of conventional TCR-T and IL-21R-TCR-T after 36 h coculture with HepG2 was measured by LDH assay (*n* = 4). **f** The supernatant IFN-γ levels of TCR-T or IL-21R-TCR-T during multiple round coculture with HepG2 were measured by ELISA (*n* = 4). **g** Schematics of the subcutaneous HepG2 model establishment and different TCR-T transferring in NPG mice. The tumor volume and TCR-T proportion in mice peripheral blood were monitored until the end of the experiment. **h** The mice tumor volume in each group after HepG2 implantation and mock-transduced T cells, TCR-T or IL-21R-TCR-T transfer was shown (*n* = 5). **i** The subcutaneous HepG2 tumor was isolated from NPG mice in each group at the end of the experiment, and the weight of isolated tumors was shown (*n* = 5). **j** The percentage of TCR^+^ T cells in total lymphocytes (mice and human lymphocytes) in the peripheral blood of each mouse 1, 7 and 14 days after TCR-T transfer was shown (*n* = 5). **k** NPG mice were subcutaneously injected with HepG2 and transferred with TCR-T or IL-21R-TCR-T. Mice were sacrificed 7 days after transferring and the cells in the tumor, peripheral blood, bone marrow and spleen were analyzed. **l** The numbers of tumor-infiltrating CD8^+^ TCR-T in each mouse were measured by flow cytometry and shown (*n* = 5). **m** The percentage of TCR^+^ T cells in tumor-infiltrating lymphocytes of each mouse 7 days after TCR-T transfer was shown (*n* = 5). **n** The TCR-T percentage in conventional TCR-T and IL-21R-TCR-T before and after 7 days of transfer was measured by flow cytometry. **o** The fold change of CD8^+^ TCR-T percentage in peripheral blood and tumor 7 days after transfer was shown (*n* = 5). Data were shown as mean ± SEM, *P < 0.05, **P < 0.01, ***P < 0.001, ****P < 0.0001, NS not significant, ACT adoptive cell transfer

Since our previous results showed that exogenous IL-21 boosted the proliferation of AFP-TCR-T after activation, we investigated whether IL-21R-TCR-T possesses superior proliferation capacity. The cell growth of IL-21R-TCR-T was monitored during our expanding procedure (Supplementary Fig. 4a). We found that the cell growth of CD8^+^ IL-21R-TCR-T was comparable to TCR-T cells in the CD8^+^ population after the first 16 days of CD3/CD28 activation and lentivirus transduced (Supplementary Fig. 4b). After receiving the second CD3/CD28 activation (Fig. 5a), IL-21R-TCR-T showed better proliferation capacity than conventional TCR-T cells in CD8^+^ population as the fold change of cell growth in the CD8^+^ population of IL-21R-TCR-T was almost twice as much as conventional TCR-T after second CD3/CD28 activation at day 24 (Fig. 5b). However, the CD4^+^ population of IL-21R-TCR-T showed compromised proliferation capacity during expanding (Supplementary Fig. 4b, d), which was contrary to the CD8 population. The proliferation of IL-21R-TCR-T after tumor stimulation was also monitored. We found that the percentage of divided cells was significantly higher in IL-21R-TCR-T than conventional TCR-T after 96 h coculture with HepG2 (Supplementary Fig. 4e), demonstrating the superior proliferation capacity of IL-21R-TCR-T. After three rounds repetitive HepG2 stimulation, the percentage of CD8^+^ IL-21R-TCR-T showed more increase than conventional TCR-T (Fig. 5c). These results suggested that the IL-21 signal from engineered IL-21R promoted the activation-induced proliferation in CD8^+^ TCR-T.

The antitumor function of IL-21R-TCR-T was subsequently evaluated by repetitive HepG2 stimulation assay. Under the microscope, we found that IL-21R-TCR-T showed superior killing activity during the process of repetitive HepG2 coculture compared with conventional TCR-T (Fig. 5d). The quantified killing rate showed that IL-21R- TCR-T maintained its killing function with approximately 80% HepG2 killed even at the Round 3 coculture, whereas the killing rate of conventional TCR-T gradually decreased to only 20% in the Round 3 coculture (Fig. 5e). Superior IFN-γ secretion activity was also found in IL-21R-TCR-T compared with conventional TCR-T, although both were compromised at Round 2 and 3 coculture with HepG2 (Fig. 5f). The constitutive signaling and augmented killing activity of IL-21R-TCR-T was abrogated by STAT3 blocking using STAT3 inhibitor “Stattic” (Supplementary Fig. 3d, e). Besides, CD34-TCR-T showed comparable killing activity to conventional TCR-T (Supplementary Fig. 5c, d). These results indicated the augmented antitumor function of IL-21R-TCR-T was related to the STAT3 signaling transmitted by the IL-21R dimer instead of the expression of CD34 ectodomain.

The in vivo antitumor capacity of IL-21R-TCR-T was investigated using HepG2 subcutaneous model in NPG mice (Fig. 5g). We found that the tumor growth was inhibited by both conventional TCR-T and IL-21R-TCR-T treatment and was persistently inhibited in mice receiving IL-21R-TCR-T (Fig. 5h), demonstrating the robust long-lasting in vivo antitumor efficacy of IL-21R-TCR-T. The weight and picture of tumors isolated at the end of the experiment (Fig. 5i and Supplementary Fig. 5f) also showed IL-21R-TCR-T treatment achieved better tumor control than conventional TCR-T. Besides, the tumor growth in mice that received CD34-TCR-T was comparable to conventional TCR-T (Supplementary Fig. 5e), indicating that the constitutive IL-21 signal rather than CD34 ectodomain led to the superior antitumor function of IL-21R-TCR-T. Meanwhile, the proportion of TCR-T in mice peripheral blood after transfer was also monitored. We found that the TCR-T percentage was higher in mice receiving IL-21R-TCR-T treatment than conventional TCR-T (Fig. 5j) after 7 days and 14 days of transfer, suggesting that engineered IL-21R expression enhanced the in vivo persistence of TCR-T. To investigate the tissue distribution of transferred T cells, tumor-bearing mice were sacrificed at day 7 after TCR-T (with low TCR^+^ percentage) transferring (Fig. 5k). We found that the TCR-T percentage in tumor-infiltrating lymphocytes was significantly higher in mice receiving IL-21R-TCR-T transfer than conventional TCR-T after cell transfer (Fig. 5m). The amounts of tumor-infiltrating CD8^+^ and CD4^+^ TCR-T were both higher in mice receiving IL-21R-TCR-T than conventional TCR-T (Fig. 5l and Supplementary Fig. 9a), especially in the CD8^+^ TCR-T subsets with an approximately ten-fold increase in mice receiving IL-21R-TCR-T treatment. Meanwhile, the percentage of CD8^+^ TCR-T rather than CD4^+^ TCR-T showed more increase within the tumor in mice receiving IL-21R-TCR-T than conventional TCR-T treatment (Fig. 5n, o and Supplementary Fig. 9b), demonstrating the superior proliferation and infiltrating capacity of CD8^+^ IL-21R-TCR-T within the tumor microenvironment. The percentage of CD4^+^ TCR-T subsets in mice peripheral blood was higher in IL-21R-TCR-T 7 and 14 days after transfer (Supplementary Fig. 9e), indicating that the IL-21R promoted the persistence of CD4^+^ TCR-T, which led to the increased amounts of tumor-infiltrating CD4^+^ TCR-T cells (Supplementary Fig. 9a). The transferred cells and TCR^+^ cells percentage in the bone marrow and spleen 7 days after transfer was comparable in mice receiving TCR-T and IL-21R-TCR-T (Supplementary Fig. 9c, d), indicating that the powerful infiltrating capacity of IL-21R-TCR-T depends on the presence of tumor antigen.

### **Engineered IL-21R promoted naive-like phenotype differentiation and alleviated apoptosis and exhaustion in TCR-T**

**Fig. 6:**
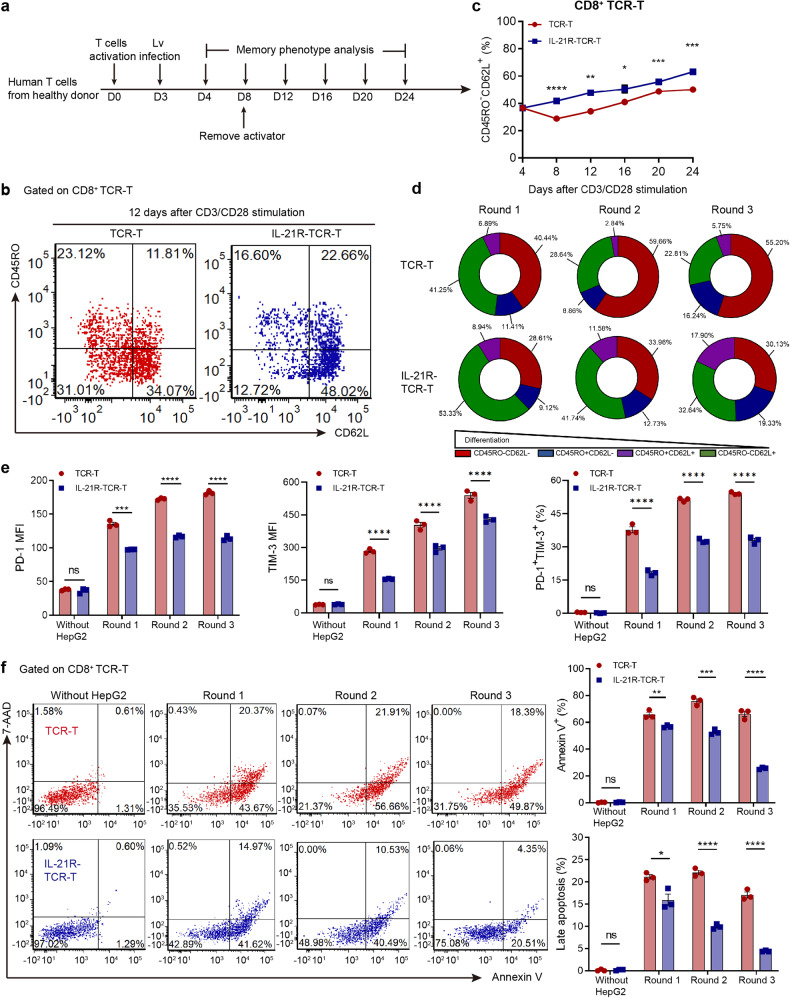
IL-21R-TCR-T maintained stemness and showed less exhausted phenotype and apoptosis after CD3/CD28 or tumor stimulation. **a** The memory phenotype of TCR-T was monitored during the ex vivo generation process every 4 days. **b** The CD45RO and CD62L expression of CD8^+^ TCR-T and IL-21R-TCR-T 12 days after CD3/CD28 activation measured by flow cytometry was shown. **c** The percentage of CD45RO^-^CD62L^+^ population in CD8^+^ TCR-T or IL-21R-TCR-T from Day 4 to Day 24 after CD3/CD28 activation was shown respectively (*n* = 3). **d** The CD45RO and CD62L expression of the CD8^+^ TCR-T and IL-21R-TCR-T was measured by flow cytometry after 36 h coculture with HepG2. The percentage of different subpopulations determined by CD45RO and CD62L in CD8^+^ TCR-T and IL-21R-TCR-T after 36 h coculture with HepG2 was shown. **e** The PD-1 and TIM-3 expression of CD8^+^ TCR-T or IL-21R-TCR-T after each round cocultured with HepG2 was measured by flow cytometry. The percentage of PD-1^+^TIM-3^+^ proportion and MFI of PD-1 and TIM-3 in CD8^+^ TCR-T was shown (*n* = 3). **f** The viability of CD8^+^ TCR-T and IL-21R-TCR-T after 36 h coculture with HepG2 was measured by flow cytometry. The percentage of Annexin V positive and late apoptosis cells (7-AAD^+^) was shown (*n* = 3). Data were shown as mean ± SEM, *P < 0.05, **P < 0.01, ***P < 0.001, ****P < 0.0001, NS not significant

We then investigated the phenotypic change bought from the constitutive IL-21 signal in IL-21R-TCR-T. The memory phenotype of IL-21R-TCR-T was monitored during the ex vivo expanding process (Fig. 6a). We found that IL-21R-TCR-T maintained a higher percentage of CD45RO^-^CD62L^+^ naive-like population than conventional TCR-T, especially in the CD8 subset after CD3/CD28 activation (Fig. 6b, c). CD4^+^ IL-21R-TCR-T also maintained a higher percentage of CD45RO^-^CD62L^+^ subset during the expansion phase (Supplementary Fig. 4c) despite the weaker proliferation capacity. CD34-TCR-T showed comparable CD45RO and CD62L expression to conventional TCR-T during the expansion phase (Supplementary Fig. 5a, b), indicating that the constitutive IL-21 signal induced the naive-like phenotype instead of the expression of CD34 ectodomain in IL-21R-TCR-T. The phenotypic alteration during repetitive HepG2 stimulation in IL-21R-TCR-T was also monitored. IL-21R-TCR-T showed a higher percentage of naive-T cells (CD45RO^−^CD62L^+^) and central memory T cells (CD45RO^+^CD62L^+^) than conventional TCR-T after each round coculture with HepG2 (Fig. 6d and Supplementary Fig. 6c). Meanwhile, the PD-1 and TIM-3 expression and percentage of PD-1^+^TIM-3^+^ subsets were lower in CD8^+^ IL-21R-TCR-T than conventional TCR-T during repetitive coculture with HepG2 (Fig. 6e and Supplementary Fig. 6a, b), indicating that engineered IL-21R expression alleviated the exhaustion of TCR-T during repetitive tumor antigen stimulation. The percentage of Annexin V^+^ and 7-AAD^+^ population was lower in IL-21R-TCR-T than conventional TCR-T (Fig. 6f), suggesting that engineered IL-21R expression prevented the apoptosis of TCR-T during repetitive tumor antigen stimulation.

### **Single-cell RNA sequencing (scRNA-seq) further revealed the immunological characteristics of IL-21R-TCR-T**

**Fig. 7:**
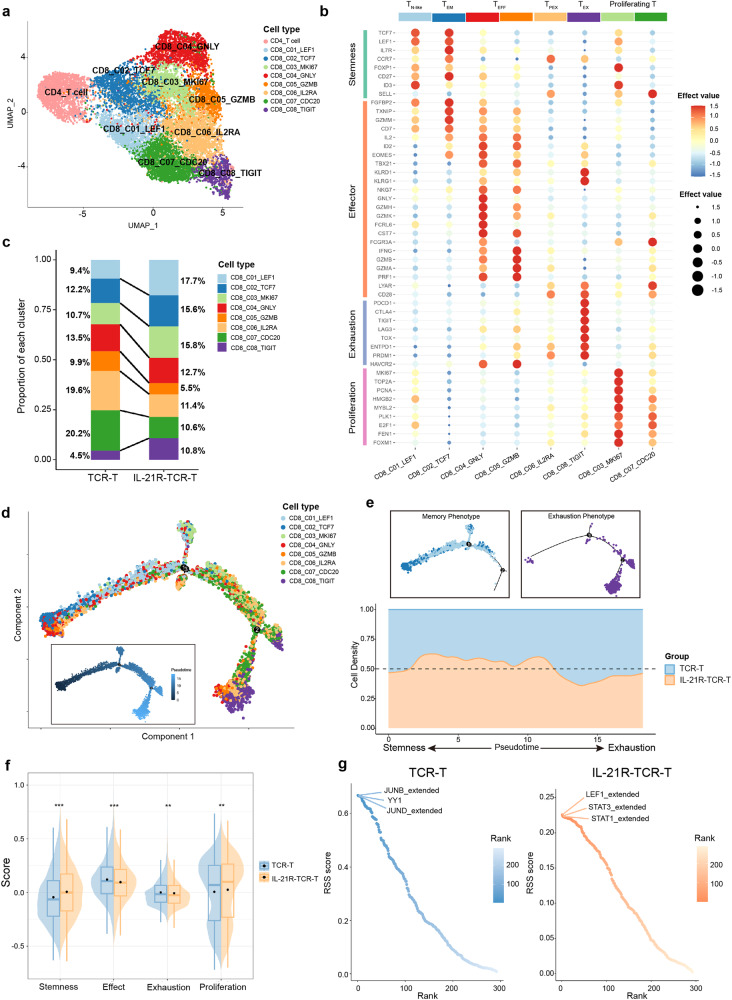
Immunological characteristics of IL-21R-TCR-T profiled by scRNA-seq. **a** Combined UMAP (Uniform Manifold Approximation and Projection) plot of all sequenced cells that passed QC (Quality Control) for subsequent analyses in this study. The color denotes cell types. **b** Characteristics of the CD8^+^ T cells clusters as defined by the expression of a panel of functional properties relevant genes. Both color and size indicate the effect value. **c** Barplot showing the contribution of cell types to each group. **d** Monocle2 trajectory plot of CD8^+^ T cells. Cell orders are inferred from the expression of highly variable genes except cell cycle-related genes. Color is coded by CD8^+^ T cell subpopulations. Insert visualizes the pseudotime defined by Monocle2. **e** Top, respectively showing memory CD8^+^ T cells (left) and exhausted T cells (right) in (**d**). Bottom, cell density of TCR-T and IL-21R-TCR-T along with the pseudotime defined by Monocle 2. **f** Split violin plot showing the distribution of several immune-related scores of T cells. **P < 0.01, ***P < 0.001, ****P < 0.0001, Mann–Whitney test was used to calculate the significance. The black dots represent the mean score. **g** Left, Rank for regulons in TCR-T based on regulon specificity score (RSS). The top three regulons are highlighted. Right, same for IL-21R-TCR-T

In the final part, Single-cell sequencing technology was applied to demonstrate the immunological features of IL-21R-TCR-T. Conventional TCR-T and IL-21R-TCR-T were isolated after coculture with HepG2, and their transcriptomes were profiled at the single-cell level (Supplementary Fig. 7a, b). After quality control, 12691 cells were kept and subsequently divided into eight CD8^+^ and one CD4^+^ cluster (Supplementary Fig. 7c, d and Fig. 7a). As CD8^+^ T cells are responsible for the main tumor-killing effect in T cell therapy, we further concentrated on the functional annotation of CD8^+^ T cell subpopulations according to their distinct signature gene expression (Fig. 7b, Supplementary Fig. 8a, b and Supplementary Table S1). CD8_C01_LEF1 was identified as naive-like T cells with the highest stemness score. CD8_C02_TCF7 was designated as T_EM_ due to the higher effector T cell properties than CD8_C01_LEF1. Within the two effector T cell subsets, CD8_C04_GNLY produced *GNLY*, *GZMH* and *GZMK* effector modules, whereas CD8_C05_GZMB highly expressed typical effect genes, such as *GZMB*, *GZMA* and *PRF1*. Both CD8_C08_TIGIT and CD8_C06_IL2RA were annotated as dysfunctional T cells. CD8_C08_TIGIT was characterized as terminally exhausted subsets based on the upregulated exhaustion markers. CD8_C06_IL2RA showed a low expression of stemness and effector signature and moderate exhaustion property, corresponding to the transitional stage between effector T cells and terminal exhausted T cells, and thus was defined as pre-exhausted T cells (T_PEX_). We also identified two proliferating T cells with extremely high proliferation scores and predominantly in the active phase of cell proliferation (Supplementary Fig. 7e, f). In comparison with the CD8_C07_CDC20 cluster, the CD8_C03_MKI67 cluster showed higher expression of proliferation-related and stemness-related genes and shared the highest Jaccard similarity with CD8_C01_LEF1 and CD8_C02_TCF7 compared other clusters, probably representing high-proliferative memory T cells. CD8_C07_CDC20 was most similar to the T_PEX_, thus preferentially designated as high-proliferative T_PEX_ (Fig. 7b and Supplementary Table S3).

Given the detailed cell annotation, we compared the distinct functional phenotype preferences exhibited in IL-21R-TCR-T and conventional TCR-T. Almost half of IL-21R-TCR-T (49.1%) preferentially exhibited memory properties, much higher than the fraction of memory T cells in conventional TCR-T (32.3%), confirming that the engineered IL-21R induced memory differentiation in TCR-T. Moreover, IL-21R-TCR-T showed a lower percentage of dysfunctional T cell subsets (T_PEX_ + T_EX_) in comparison with TCR-T (Fig. 7c). Even if our previous experiments have demonstrated the superior proliferation capacity of IL-21R-TCR-T upon CD3/CD28 activation, the proportion analysis supported by scRNA-seq data further revealed the phenotype of proliferating T cells after tumor antigen stimulation in detail. We found that high-proliferative T_EM_ comprised a larger population in proliferating T cells in IL-21R-TCR-T instead of high-proliferative T_PEX_, whereas proliferating TCR-T contained more high-proliferative T_PEX_ (Fig. 7c). Additionally, Monocle trajectory analysis showed the transition from CD8_C01_LEF1 and CD8_C02_TCF7 to CD8_C08_TIGIT, exactly reflecting the developmental trajectory from memory T cells to terminal exhausted T cells with increased exhaustion score and decreased stemness and effector score (Fig. 7d and Supplementary Fig. 8c). Along the differentiation trajectory, IL-21R-TCR-T tended to accumulate in the early stage with heightened stemness, whereas conventional TCR-T tended to accumulate in the late developmental trajectory, indicating that IL-21R-TCR-T were prone to differentiate into stem-cell-like and memory phenotype subsets after sustained tumor antigen exposure (Fig. 7e). Then, an immune-related scoring algorithm was applied to quantify the immunological features. As expected, IL-21R-TCR-T exhibited profoundly higher stemness and proliferation scores and relatively lower exhaustion scores in contrast to TCR-T (Fig. 7f). Altogether, these results indicated that the engineered IL-21R boosted T cell stemness and proliferation capacity and prevented exhaustion during continuous antigen challenge, resulting in the prolonged and augmented antitumor efficacy of IL-21R-TCR-T.

We next analyzed the differentially expressed genes between IL-21R-TCR-T and conventional TCR-T. In contrast to conventional TCR-T, IL-21R-TCR-T highly expressed T cell stemness and activation-related genes, including *TCF7*, *LEF1*, *XCL1* and *GZMM*. On the other hand, IL-21R-TCR-T exhibited lower levels of *IL-10* and *PIM2* gene expression, which is correlated with the immunosuppression program^27^ (Supplementary Fig. 8d). Notably, a recent study uncovered that osteopontin (OPN) (encoded by *SPP1*) would activate CD29 on tumor-reactive CD8^+^ T cells and result in TOX-mediated CD8^+^ T cells dysfunction.^28^ Accordingly, *SPP1* was significantly downregulated in IL-21R-TCR-T, which may contribute to the lower dysfunctional T cell subsets proportion in IL-21R-TCR-T (Supplementary Fig. 8d). To further demonstrate the functions and immunological roles of these differentially expressed genes, we performed GO term enrichment analysis and found that these genes are significantly enriched in lymphocyte differentiation and T cell activation regulation (Supplementary Fig. 8e).

Focusing on the differences in regulon activity between IL-21R-TCR-T and conventional TCR-T, we found that several JUN family members exhibited high regulon specificity in TCR-T. *LEF1*, *STAT3* and *STAT1* were inferred as essential regulators for IL-21R-TCR-T by SCENIC analysis (Fig. 7g and Supplementary Table S4). Among them, *LEF1* is required for the stemness of CD8^+^ T cells,^29,30^ which may play a crucial role in maintaining the stemness subsets in IL-21R-TCR-T. Besides, our data showed elevated STAT3 transcriptional activity in IL-21R-TCR-T and corroborated the constitutive IL-21 signaling in IL-21R-TCR-T.

## Discussion

In this study, IL-21 was identified as a potent cytokine for augmenting the antitumor capabilities of TCR-T and a novel IL-21 receptor with constitutive phosphorylated STAT3 signaling was established. Phenotypic analysis showed that the constitutive IL-21 signal from engineered IL-21R endowed TCR-T with a naive-like phenotype with upregulation of stemness-related genes and alleviated apoptosis and exhaustion of TCR-T during repetitive antigen exposure. The superior antitumor function of IL-21R-TCR-T was verified in repetitive coculture assay and xenograft HCC model. Adoptive T cell therapy emerges as a promising therapeutic avenue for hepatocellular carcinoma (HCC) patients, capitalizing on the abundance of antigens, including tumor-associated antigens, cancer-testis antigens, and viral-derived antigens^7^ within HCC tumor cells. Preliminary clinical trial data of HCC adoptive T cell therapy showed considerable antitumor activity of CAR-T cells targeting CD133 and GPC3,^31,32^ highlighting the prominent therapeutic value of adoptive T cell therapy in HCC patients. However, the HCC-specific microenvironment restricts the access of transferred T cells to tumor cells and impairs the cytotoxicity of tumor-infiltrating T cells,^7^ thus limiting the efficacy of adoptive T cell therapy in HCC patients. In this study, we demonstrated that the IL-21 signal is a promising target for improving the therapeutic efficacy of AFP-TCR-T in HCC treatment. The IL-21R-TCR-T showed superior antitumor functionality and remarkable proliferation and infiltrating capacity within the HCC microenvironment. These findings suggest that harnessing the IL-21 signal presents an optional strategy to surmount obstacles imposed by the HCC microenvironment in adoptive T cell therapy.

IL-21 is primarily produced by CD4^+^ T cells such as T follicular helper cells and Th17 cells^12^ and has been reported to preserve T cells in a less differentiated state with the expression of stem-cell-related transcription factors such as *TCF7* and *LEF1*.^18,33^ T cells undergoing expansion solely with IL-21 exhibited diminished proliferation compared to those with IL-2.^18^ This observation implies the existence of divergent biological programs regulated by IL-2 and IL-21. In our study, we found that IL-21 synergized with IL-2 not only augmented the expansion of TCR-T but also retained its biological effect to promote naive-like phenotype T cell differentiation, suggesting that IL-2 and IL-21 combination may overcome the impact of terminal effector differentiation and exhaustion bought from persistent IL-2 stimulation^34,35^ and the proliferation inhibition bought from IL-21. Except for the capacity to generate naive-like T cells, IL-21 is also required for the formation of the CX3CR1^+^ cytolytic subset, therefore promoting the cytotoxicity of CD8^+^ T cells with enhanced Granzyme B and IFN-γ expression.^35,36^ The defect in effector transition from low-differentiated memory T cell subsets inside the tumor may dampen the antitumor function of tumor-infiltrating T cells,^36^ indicating that strategies that only maintain T cells low differentiated state are unsuitable for transferred T cells inside the tumor microenvironment. In our study, we found that the IL-21-supplemented TCR-T showed a signature of naive-like T cells after activation and simultaneously retained its effector function against tumor cells, reflecting the unique biological effects of IL-21 in supporting T cell antitumor immunity. Consequently, the constitutive STAT3 signaling imparted by the engineered IL-21R induced a unique phenotypic alteration in IL-21R-TCR-T, resulting in a “long-lived effector” phenotype following tumor stimulation. This distinctive phenotype endowed IL-21R-TCR-T with superior antitumor function and enhanced proliferation capacity upon activation.

The STAT3 signaling transferred by the engineered IL-21R composed of IL-21Rα chain homodimer was weaker than natural IL-21 receptors in our TCR-T (Supplementary Fig. 3b, c). The differences may result from the deficiency of γ chain that activated JAK3 downstream in the engineered IL-21 receptor, which only activated JAK1 downstream^12^ through the IL-21Rα chain. The attenuating signal was similar to the gain-of-function mutation of IL-7 receptor α chain (IL-7Rα) that led to the dimerization of IL-7Rα in some lymphoblastic leukemias patients.^37,38^ STAT3 is pivotal in developing terminally differentiated effector CD8^+^ T cells by facilitating the expression of genes associated with effector functions.^39^ Consequently, sustained activation of STAT3 may potentially induce terminal differentiation and exhaustion in T cells. Our study demonstrates that even attenuated IL-21 signaling was sufficient to modulate the phenotype and enhance the antitumor function of TCR-T. Since the IL-7R dimer with constitutive signaling came from lymphoblastic leukemias,^37^ the utilization of the structure like IL-7R dimer should be cautious. The constitutive IL-7 signal transferred by mutant IL-7Rα was applied in CAR-T cell therapy, which increased the proliferation, survival and antitumor activity of GD2-CAR-T cells.^37,40^ T cells with IL-7R dimer can be deleted by the iC9 suicide system^40^ for the safety of IL-7R dimer utilization. The native IL-21 signaling showed different biological effects from the IL-7 signaling. T cells with IL-21 signaling alone showed dampened proliferation capacity in vitro,^18^ indicating the great safety of the IL-21R dimer utilization.

The proliferation of CD4^+^ TCR-T was compromised by the constitutive IL-21 signal from engineered IL-21R in our study. CD4^+^ T cells play a substantial role in supporting CD8^+^ T cell function and have been used by many investigators in adoptive therapy.^41^ However, the CD8^+^ subsets in our AFP-TCR-T system are the main effectors that can kill the tumor cells instead of the CD4^+^ TCR-T subset.^42^ The potential defect in CD4^+^ TCR-T does not impair the effector function of CD8^+^ TCR-T subsets against tumors in our study. We think it may be the different signaling between native IL-21 and our engineered IL-21R (lack of γ chain signaling) that led to the impaired proliferation of CD4^+^ T in vitro. Despite the impaired in vitro proliferation, our IL-21R enhanced the persistence of CD4^+^ TCR-T in vivo (Supplementary Fig. 9e), which led to increased tumor-infiltrating cells.

Overall, our study showed that the IL-21 signal enhanced the antitumor function of AFP-TCR-T in multiple aspects. AFP-TCR-T expressing a novel engineered IL-21R with constitutive IL-21 signal showed increased proliferation and tumor-infiltrating capacity, resistance to antigen-induced cell death, and superior antitumor function compared with conventional AFP-TCR-T. However, the in vivo antitumor efficacy of IL-21R-TCR-T was only validated in a relatively artificial HCC subcutaneous model in our study since no TCR-T therapy against orthotopic HCC model was reported until now. The efficacy of engineered IL-21R within the orthotopic tumor model and other engineered T cell therapy needs further investigation. Our study underscores the effectiveness of AFP-TCR-T armed with an engineered IL-21 receptor as a promising strategy in combating HCC. We posit that the engineered IL-21 receptor represents a potent approach to enhance the efficacy of transferred T cells against various solid tumors.

## Materials and methods

### Mice

Immunocompromised NPG mice (female 7–10 weeks) were obtained from the Beijing Vitalstar Biotechnology Company and maintained in a specific pathogen-free facility at Nanfang Hospital. Animal protocols (IACUC-LAC-20230717-001) were approved by the Institutional Animal Care and Use Committee of Nanfang Hospital.

### Cell lines

Jurkat cells were obtained from iCell Bioscience Inc (Shanghai, China). Human HLA-A2^+^/AFP^+^ HepG2 cell lines were obtained from iCell Bioscience Inc and the expression of AFP and HLA-A2 was verified by western blot and flow cytometry. Jurkat T lymphocytes were cultured in RPMI 1640 medium (Gibco, Carlsbad, CA, USA) supplemented with 10% heated fetal bovine serum (Gibco), 100 U/mL penicillin (Gibco), and 0.1 mg/mL streptomycin (Gibco). HepG2 cell lines were cultured in completed Dulbecco’s Modified Eagle’s Medium (DMEM) (Gibco) supplemented with 10% unheated fetal bovine serum (Gibco), 100 U/mL penicillin (Gibco) and 0.1 mg/mL streptomycin (Gibco). All cells were cultured at 37 °C in a humidified atmosphere incubator containing 5% CO_2_.

### Construction of xenograft models, adoptive T cell transfer, and tumor-infiltrating T cell analysis

For the construction of the xenograft tumor model, five million HepG2 cells were subcutaneously inoculated into the flank of NPG mice. Each time, one vial of HepG2 cells was thawed and used for less than six passages to maintain their authenticity. After 9–12 days, the indicated numbers of human TCR-T (five million TCR^+^ cells in Fig. 2f and Fig. 5g, three million TCR^+^ cells in Fig. 5k) or mock-transduced T cells supplemented with 5 × 10^4^ U human recombinant IL-2 were intravenously transferred into the immunocompromised mice bearing human HepG2 tumors. TCR-T cells in mice blood were monitored by immunological staining. Tumor growth was monitored by measuring the length, width, and height three times a week. Tumor volume was calculated as 1/2 (length × width × height). At the end of the experiment, the mice were euthanized and the excised tumors were weighed and photographed. To detect tumor-infiltrating human TCR-T, the excised tumors were dissociated using Tumor Dissociation Kit (Human, Miltenyi Biotec, Bergisch Gladbach, Germany) following the instructions and analyzed by flow cytometry.

### Generation of recombinant lentivirus

The protein sequences of human CD34 IL-21R and IL-7R were obtained from the NCBI database. The mutant IL-21 receptor was constructed by the extracellular domain of CD34, the transmembrane domain of IL-7R with a CPT insertion between amino acids 245 and 246, the intracellular domain of IL-21R. CD34 was constructed by the extracellular domain of CD34, the transmembrane domain of IL-7R with a CPT insertion between amino acids 245 and 246. The protein sequences were converted to the gene sequences. The genes were codon-optimized, synthesized, and cloned into lentivirus expressing AFP-specific TCR.^42^ The sequence of AFP-TCR can be found in Supplementary Fig. 11, the ref. ^43^ and the patent (CN110662760A) at China National Intellectual Property Administration. Lentivirus was prepared and titrated by Genscript (Shanghai, China).

### Transduction and in vitro expansion of human T cells

Human T cells were isolated from the buffy coat of healthy donors by negative selection and activated by the CD3/CD28 tetrameric antibody complex (Stemcell Technologies, Vancouver, Canada) for three days before transducing with lentivirus. The CD3/CD28 antibody complex was rinsed away four days after lv transduction. 10 ng/ml of human recombinant interleukin-2 (rIL-2, PeproTech, Rocky Hill, NJ, USA) was used in the culture system through the process. T cells were cultured in RPMI 1640 medium (Gibco) supplemented with 10% heated fetal bovine serum (Gibco), 100 U/mL penicillin (Gibco) and 0.1 mg/mL streptomycin (Gibco), 10 mM HEPAS (Gibco), 1× MEM NEAA (Gibco) and 55 μM 2-Mercaptoethanol (Gibco). When the cell density exceeds 2.5 × 10^6^ cells/ml or the medium turns yellow, split cultures back to a density of 0.5–1 × 10^6^ cells/ml in a culture medium containing 10 ng/ml rIL-2.

### Flow-cytometry analysis and antibodies

The antibodies used in this study include antibodies to human CD8 (Clone: SK1, BioLegend, San Diego, CA, USA), CD4 (Clone: RPA-T4, BioLegend), CD45 (Clone: 2D1, BioLegend), CD45RO (Clone: UCHL1, BioLegend), CD360 (IL-21Rα, clone: 2SX21R, Invitrogen, Carlsbad, CA, USA), CD62L (Clone: DREG-56, BioLegend), PD-1 (Clone: EH12.2H7, BioLegend), TIM-3 (Clone: F38-2E2, BioLegend), CD34 (Clone: 561, BioLegend), mouse TCRVβ8.3 (clone: H57-597, BioLegend), Annexin V (BioLegend), 7-AAD (BioLegend), phosphorylated STAT3 (Tyr705. Clone: 13A3-1, BioLegend) and mouse CD45.2 (Clone: 104, BioLegend).

For cell surface staining, single-cell suspensions were prepared and stained with specific antibodies for 15 min at RT. Cells were washed once by phosphate buffered saline (PBS) before analysis. For Annexin V staining, cells were resuspended in Annexin-V Binding Buffer (BioLegend) and stained with antibodies for 30 min at 4 °C. For intracellular staining of phosphorylated STAT3, cells were rested in the completed medium without interleukins for 3 h at 37 °C, and then IL-21 was added into the medium or not, followed by fixation and permeabilization using the Fixation Buffer (BioLegend) and Perm Buffer (BioLegend) and stained with specific antibodies for 60 min on ice. Before analysis, cells were washed once with the Cell Staining buffer (BioLegend). All the stained samples were analyzed in a BD FACS canto II and the flow cytometric data were analyzed using FCS Express VI software.

### In vitro repeated coculture assay

HepG2 cells (1 × 10^5^) were seeded into 96-well culture plates for 24 h, after which TCR-T (effector to target ratio = 0.3:1, the effector refers to AFP-TCR positive cells) were transferred into the 96-well plate in the presence of recombinant human IL-2 (10 ng/ml, PeproTech) with or without IL-21 (20 ng/ml, PeproTech), IL-7 (20 ng/ml, PeproTech) and IL-15 (20 ng/ml, PeproTech) respectively. For the comparison of different lentivirus transfected TCR-T (IL-21R-TCR-T and CD34-TCR-T), the transfection rate (percentage of AFP-TCR positive cells) among groups was adjusted to a comparable level before coculture with HepG2 cells. In repetitive coculture assay, T cells after 36 h coculture (round 1) were collected and washed with PBS, resuspended in fresh medium and added to a new plate seeded with 1 × 10^5^ HepG2 cells (round 2) for another 36 h. This procedure was repeated if applicable (round 3 or more). At the end of each round, cells in duplicate wells were collected for phenotypic analysis by flow cytometry.

### Lactate dehydrogenase (LDH) assay and enzyme-linked immunosorbent assay (ELISA)

In the repetitive coculture assay, the supernatant was collected after each round of coculture for LDH or ELISA detection. The cytotoxicity of TCR-T was determined by measuring the LDH activity in the supernatant as instructed (Promega, Madison, WI, USA). ELISA detection of human IFN-γ was conducted following the instructions (BioLegend).

### T cells proliferation assay

TCR-T were labeled with Carboxyfluorescein succinimidyl amino ester (CFSE) following the instruction of CellTrace™ CFSE Cell Proliferation Kit (Thermo Fisher Scientific, Waltham, MA, USA) and cocultured with HepG2 cells (1×10^5^) at 0.3:1 E/T ratios for 4 days. Tumor cells were eradicated and TCR-T cells were collected for flow cytometry analysis.

### Western blotting analysis

For immunoblotting, protein samples from cells in each group were collected with Blue Loading Buffer Pack (7722 S, Cell Signaling Technology, Danvers, MA, USA). After heating at 100 °C for 8 min, the protein lysates were separated by sodium dodecyl sulfate-polyacrylamide gel electrophoresis (SDS-PAGE) and transferred to the PVDF membrane (Millipore, Billerica, MA, USA) to be incubated with the following primary antibodies at 4 °C overnight: anti-phosphorylated-Stat3 (Tyr705, 1:2000; 9145S; Cell Signaling Technology) and anti-β-actin antibody (1:1000; 4970L; Cell Signaling Technology). Next, the membranes were incubated with anti-rabbit or anti-mouse immunoglobulin G conjugated with horseradish peroxidase for 1 h at room temperature. Finally, the ECL detection kit (Beyotime Biotechnology, Shanghai, China) was used to detect the protein bands. The bands were photographed using the UVP BioSpectrum AC image 233 system (Upland, CA, USA).

### I**mmunohistochemistry (IHC) analysis of the subcutaneous mice tumor**

Tumors were isolated from the right flank of NPG mice 7 days after TCR-T transferring and fixed with 4% paraformaldehyde at 4 °C for 16–24 h, followed by rinsing with tap water for 3 h, dehydrating through 70%, 80%, 95%, 100% alcohol and two change of xylene and embedding with paraffin. The fixed and embedded tumors were sectioned into 4 µm sections and transferred onto glass slides at room temperature until ready for use. Sections were further deparaffinized through xylene and 100, 95, 80 and 70% alcohol and rehydrated with distilled water. Antigens were retrieved by heating the sections in EDTA (pH 9.0) and blocked with goat serum (Boster). Sections were then incubated with primary antibodies (anti-TCRβ, ab313579, Abcam, Cambridge, MA, USA) diluted 1:2000 at 4 °C overnight, stained with streptavidin-HRP and diaminobenzidine (DAB) peroxidase following the instruction of DAB Detection Kit (Gene Tech, Shanghai, China), followed by counterstaining with hematoxylin for cell nuclei. All sections were examined microscopically. The positive area was quantified by ImageJ.

### Bulk RNA sequencing

Total RNA was extracted using Trizol reagent kit (Invitrogen) and analyzed for quality on an Agilent 2100 Bioanalyzer (Agilent Technologies, Palo Alto, CA, USA). Next, the mRNA was enriched, fragmented into short fragments and reversibly transcribed into cDNA using NEBNext Ultra RNA Library Prep Kit for Illumina (New England Biolabs, Ipswich, MA, USA). Before amplification, the purified double-stranded cDNA fragments were end-repaired, base-added, ligated to Illumina sequencing adapters and then purified with the AMPure XP Beads (1.0X). Finally, the amplified cDNA library was sequenced using Illumina Novaseq6000 by Gene Denovo Biotechnology Co. (Guangzhou, China).

### Bulk RNA-Seq filtration, alignment, and quantification

Raw reads were initially filtered by fastp (version 0.18.0)^44^ and the rRNA mapped reads were further removed by the short reads alignment tool Bowtie2,^45^ which was used for mapping reads to the ribosome RNA (rRNA) database. Then, the filtered reads were mapped to the reference genome using HISAT2. 2.4^46^ and StringTie v1.3.1^47,48^ was used for the following assembly in a reference-based approach. For transcript quantification, the FPKM (fragment per kilobase of transcript per million mapped reads) value was calculated by RSEM^49^ software.

### Single-cell RNA sequencing cells preparation

HepG2 cells (2.5 × 10^5^) were seeded into 24-well culture plates for 12 h, after which TCR-T or IL-21R-TCR-T (effector to target ratio = 0.3:1, effector was defined as AFP-TCR^+^ cells) were added to the tumor cells in the presence of recombinant human IL-2 (10 ng/ml). After 36 h coculture, TCR-T or IL-21R-TCR-T were stained with TCR-PE antibody and sorted by EasySep^TM^ Release PE Positive Selection Kit (Stemcell Technologies) following the instructions.

### Single-cell RNA sequencing

ScRNA-seq libraries were prepared and sequenced by Genergy Biotechnology (Shanghai, China) Co. Ltd. according to 10× Genomics guidelines. Chromium Next GEM Single Cell 5’ Kit v2 (10× Genomics) (PN-1000263) was used to perform single-cell separation and library construction according to the manufacturer’s instructions. Briefly, the cell suspension, barcoded gel beads and partitioning oil were loaded onto the 10x Genomics Chromium Chip to generate single-cell Gel Beads-in-Emulsion (GEMs). Cell lysis and reverse transcription were performed inside individual GEMs. Then, the generated cDNA with unique cell barcodes was amplified. Next, the scRNA-seq libraries were constructed by the 5’Library Kits (PN-1000190) and sequenced on an Illumina NovaSeq 6000 platform to generate 2×150-bp paired-end reads.

### Processing and clustering scRNA-seq data

Sequencing data were aligned to the GRCh38 genome and filtered. Barcodes and unique molecular identifiers were counted using the Cell Ranger v6.1.1 command cell ranger count. Specifically, to assess the expression of the engineered IL-21R in the scRNA-seq data, the cellranger reference was reindexed (mkref) by adding a single contig for the 1889bp engineered IL-21R sequence to assembly GRCh38 of the human genome. Data were further analyzed in R by Seurat version 4.2.0.^50^ To ensure data quality, we excluded genes that were not detected in at least three cells, as well as cells with mitochondrial reads exceeding 10%. Cells expressing less than 200 genes or more than 10,000 genes were also removed. Then, data were normalized using sctransform.^51^ CellCycleScoring was used to regress out cell cycle-specific clustering using SCTransform vars.to.regress (S.Score, G2M.Score) function. In addition, IL-21R-TCR-T were identified using raw_counts [“engineered21R”,]>0.

Clusters and UMAP were generated from the first 15 PCA dimensions using the default parameter settings in Seurat. Marker genes were determined using the FindAllMarkers function in Seurat. Sctransform normalized expression was used for the heatmap of marker genes, UMAP feature plots, and dot plots. The first round of clustering was performed mainly to distinguish CD4^+^ T cells and CD8^+^ T cells. The cluster with CD4 as the signature gene was identified as the CD4^+^ T cell cluster. A second round of clustering was performed on CD8^+^ T cells to identify subpopulations with variable functional characteristics. Besides, to further identify the proliferating CD8^+^ T cell subtypes, Jaccard similarity across clusters was calculated with the top 1000 (CD8_C03_MKI67 and CD8_C07_CDC20) and top 200 cell type signature genes (other cell types).^52^

### Computing immune-related score

Stemness score, effect score, exhaustion score and proliferation score were computed by AddModuleScore function in Seurat, with corresponding marker gene sets (Supplementary Table S2). A Mann–Whitney *U* test was used to test the significance of immune-related scores between IL-21R-TCR-T and TCR-T.

### Trajectory analysis

To elucidate the potential process of T cell functional changes and determine the potential lineage differentiation between diverse CD8^+^ T subsets, we applied the Monocle (version 2) algorithm with the top 2000 highly variable genes excluding TCR genes, ribosomal gene and cell cycle-related genes, based on the rank of vst generated by Seurat.^53^ Cells were ordered through the inferred pseudotime to indicate their development trajectory.

### Analysis of DEGs (differentially expressed genes)

We identified differentially expressed genes between IL-21R-TCR-T and TCR-T clusters using DEsingle.^54^ And the list was further filtered by |log2FC | >0.25 and FDR < 0.05. Using clusterProfiler 4.7.1.003 version, gene ontology (GO) and enrichment analysis were performed for DEGs between IL-21R-TCR-T and TCR-T.^55,56^

### Identification of key transcription factors (TF) in IL-21R-TCR -T and TCR-T

SCENIC algorithm (R version) was used to identify crucial transcription factors on IL-21R-TCR-T and TCR-T.^57^ To improve running speed, we randomly sampled half of the cells from each CD8^+^ T cell cluster and the count matrix of those cells was subsequently used to infer the TF-target gene co-expression networks.^58^ Then the RcisTarget was used to identify the regulons each of which contained one TF and its target genes enriched for the motifs of the TF. The motif annotation database (hg19-500bp-upstream-7species.mc9nr.feather) and the ranking databases (hg19-tss-centered-10kb-7species.mc9nr.feather) used by the RcisTarget command were downloaded from https://resources.aertslab.org/cistarget/databases/old/homo_sapiens/hg19/refseq_r45/mc9nr/gene_based/. Next, we employed AUCell to evaluate the activities of the regulons for all CD8^+^ T cells. Given the activity scores of the regulons and the engineering type (IL-21R-TCR-T or TCR-T) assignment of the cells, the calcRSS function was used to calculate the specificity scores of the regulons (RSS). For each engineering type, the regulon with the highest RSS would be inferred as the essential regulator for the corresponding engineering type.

### Statistical analysis

Student’s *t* tests were used to determine statistically significant differences between two samples. One-way analysis of variance (ANOVA) was used to determine statistical differences among three or more groups. All statistical analyses were performed with two-tailed tests. Graph generation and statistical analyses were performed using GraphPad Prism, version 9.0 (GraphPad Software). A *P* value of less than 0.05 was considered statistically significant.

### Supplementary information

Supplementary materials

## Data Availability

All experimental data generated or analyzed during this study are available from the corresponding authors upon reasonable request. The raw sequence data reported in this paper have been deposited in the Genome Sequence Archive^59^ in National Genomics Data Center,^60^ China National Center for Bioinformation / Beijing Institute of Genomics, Chinese Academy of Sciences (GSA-Human: HRA006670 for bulk RNA-seq data, HRA006668 for single-cell RNA-seq data) at https://ngdc.cncb.ac.cn/gsa-human.
