# Supplementary material for: A novel engineered IL-21 receptor arms T-cell receptor-engineered T cells (TCR-T cells) against hepatocellular carcinoma
Source: Signal Transduct Target Ther. 2024 Apr 20;9:101. doi: 10.1038/s41392-024-01792-6 (PMC11032311; doi:10.1038/s41392-024-01792-6)
Supplement: Supplementary file 1 — Supplementary materials [file 41392_2024_1792_MOESM1_ESM.docx]

Supplementary Materials for

A novel engineered IL-21 receptor arms T-cell receptor-engineered T cells (TCR-T cells) against hepatocellular carcinoma

Wei Zhu*, Zhiming Zhang*, Jinzhang Chen*, Xiaolan Chen, Lei Huang, Xiaoyong Zhang, Xuan Huang, Na Ma, Weikang Xu, Xuan Yi, Xinyu Lu, Xin Fu, Siwei Li, Guoheng Mo, Yiyue Wang, Guosheng Yuan, Mengya Zang, Qi Li, Xiaotao Jiang, Yajing He, Sha Wu, Yukai He, Yongyin Li,†, Jinlin Hou,†

Correspondence to: [jlhousmu@163.com](mailto:jlhousmu@163.com) and [yongyinli@foxmail.com](mailto:yongyinli@foxmail.com).

*Authors share co-first authorship.

†Authors share co-corresponding authors.

**This PDF file includes:**

Figures. S1 to S11

Table S1: Overview of CD8^+^ T cell cluster characteristics

Table S2: Gene markers for computing the immune-related score

Table S3: Jaccard similarity between proliferating T-cell clusters and other clusters

Table S4: Regulon specificity score (RSS) for regulons in TCR-T cells and IL-21R-TCR-T cells

**Other Supplementary Materials for this manuscript include the following:**

None.


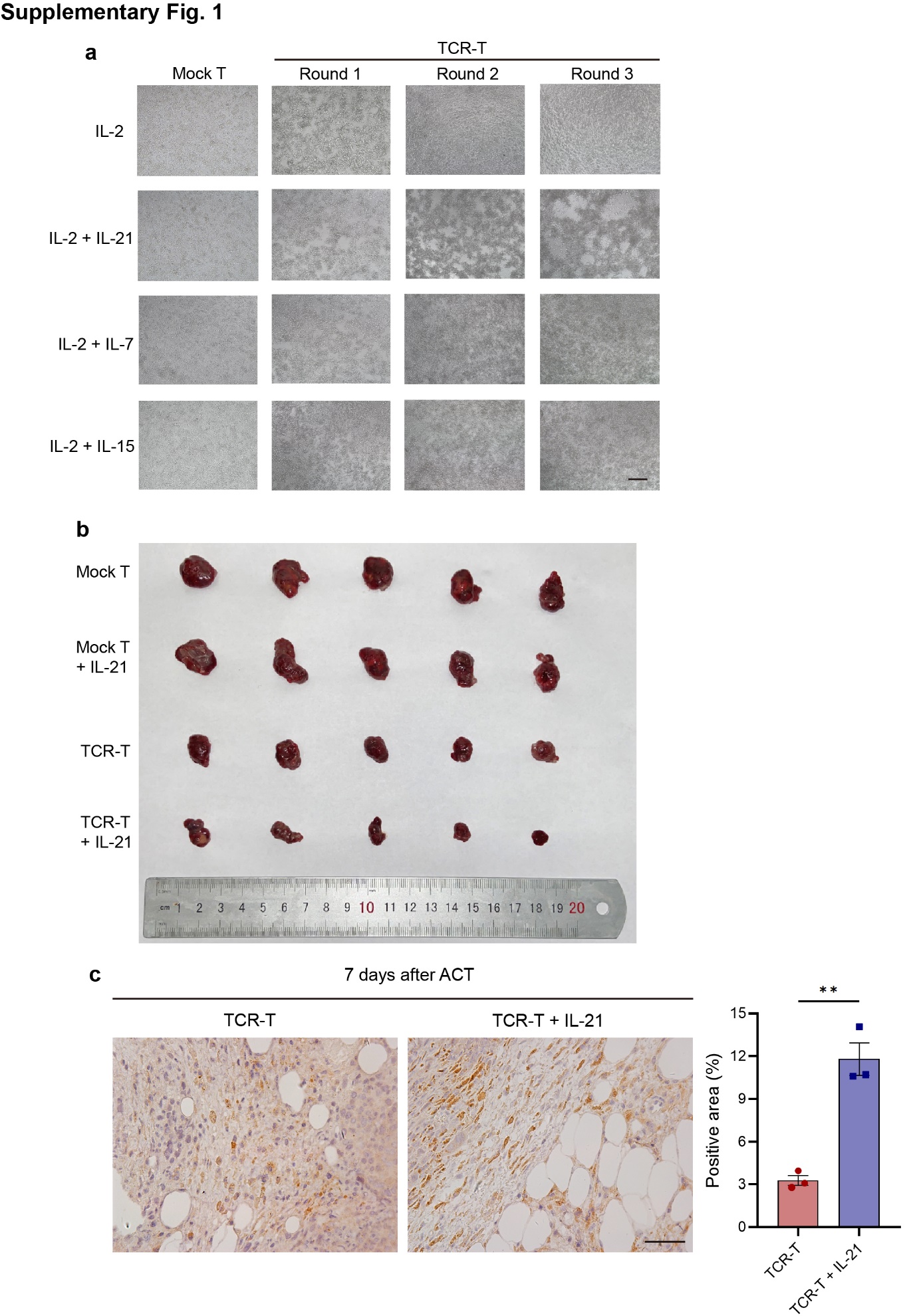


Figure. S1. TCR-T with IL-21 showed enhanced killing function and tumor infiltration. (a) Representative pictures of HepG2 were taken after 36 h cocultured with mock-transduced T or TCR-T in the presence of different cytokines. Scale bar = 100 μm. (b) Tumors were isolated at the end of the experiment from tumor-bearing mice receiving mock-transduced T or TCR-T treatment with or without IL-21. The picture of isolated tumors was shown (n = 5). (c) IHC pictures of the mice tumor 7 days after AFP-TCR-T or AFP-TCR-T with IL-21 transferring targeting the mouse TCR β chain and the quantification of the positive area in each group were shown (n = 3). Scale bar = 50μm. The data was shown as Mean ± SEM, **p <0.01.


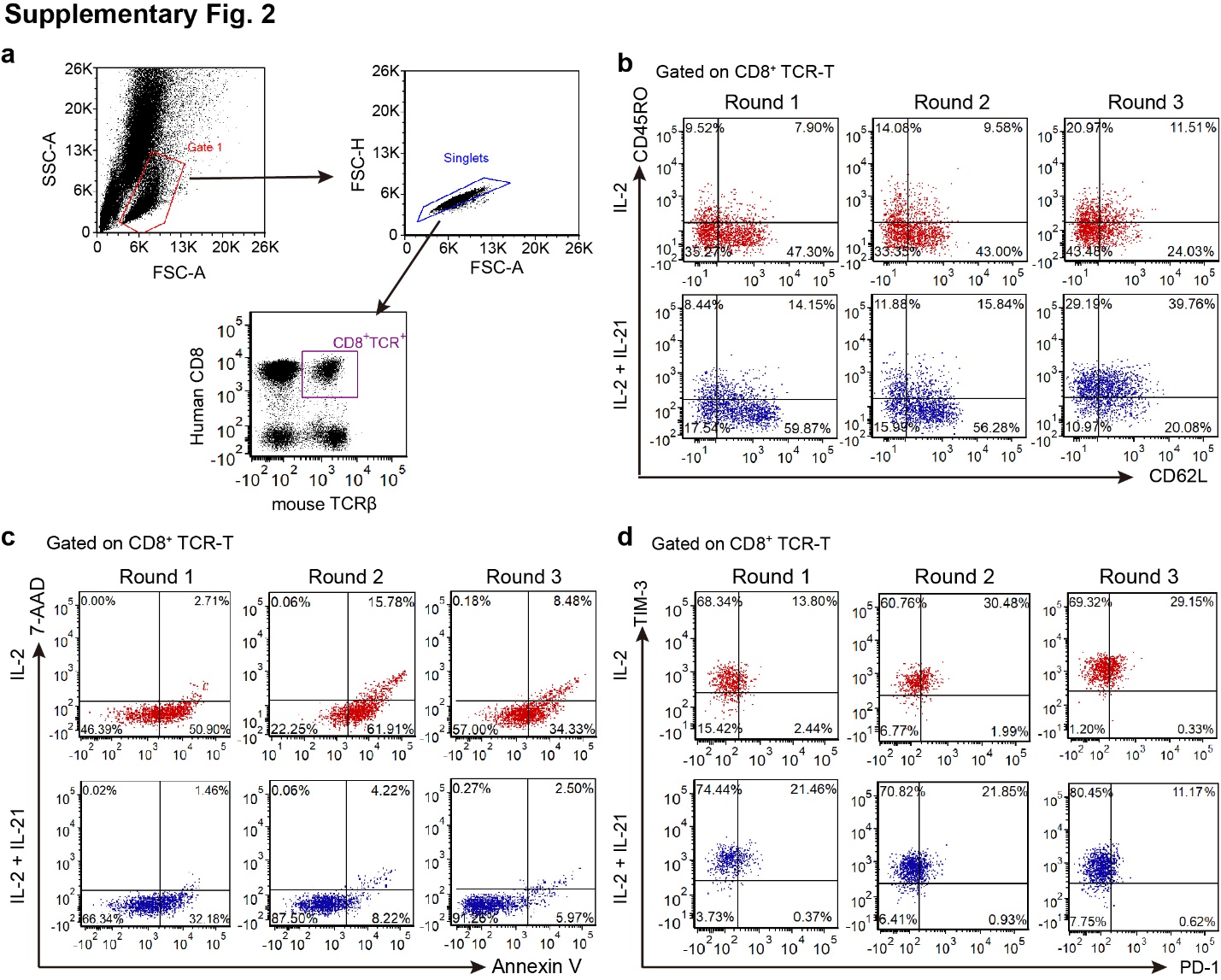


Figure. S2. IL-21-supplemented TCR-T showed increased stemness and alleviated apoptosis and exhaustion after repetitive tumor antigen stimulation. (a) The gating strategy of CD8^+^ TCR-T subsets in IL-2 or IL-21-supplemented TCR-T during repetitive coculture assay was shown. The expression of CD45RO and CD62L (b), Annexin V and 7-AAD (c), and PD-1 and TIM-3 (d) in CD8^+^ TCR-T subsets after 36h coculture with HepG2 in the presence of IL-2 or IL-2 + IL-21 was detected by flow cytometry. Representative pictures of the dot plots at each timepoint were shown.


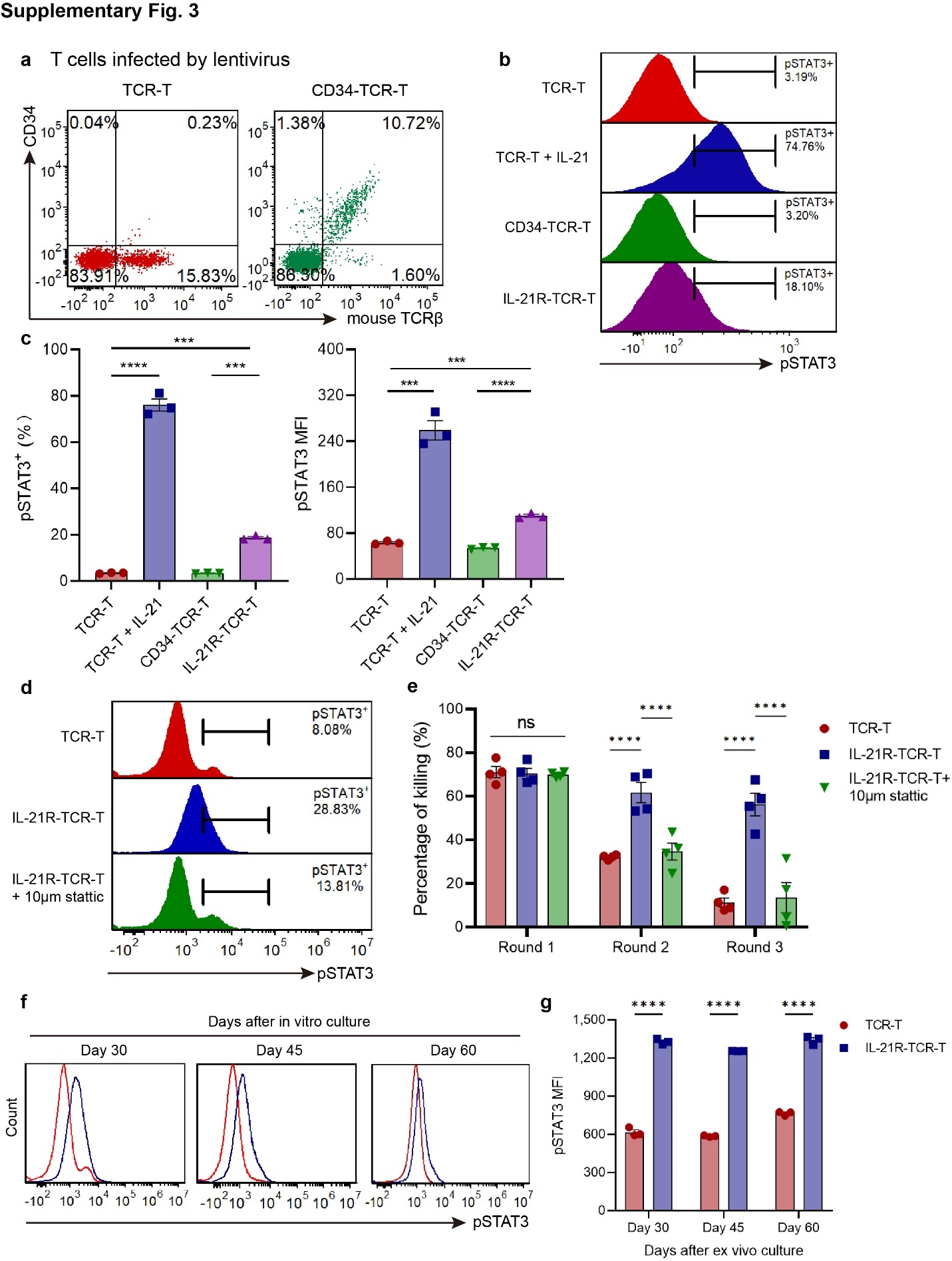


Figure. S3. Expression of the engineered IL-21R and CD34 ectodomain in CD34-TCR-T and validation of the IL-21 signal. (a) The AFP-TCR and CD34 expression in human TCR-T and CD34-TCR-T was measured by flow cytometry. The representative dot plots were shown. (b) The STAT3 phosphorylation level in human TCR-T with or without IL-21, CD34-TCR-T and IL-21R-TCR-T was measured by flow cytometry. The representative histogram of phosphorylation STAT3 expression was shown. (c) The percentage of pSTAT3^+^ cells and MFI of pSTAT3 were shown (n = 3). (d) The STAT3 phosphorylation levels in conventional TCR-T, IL-21R-TCR-T and IL-21R-TCR-T with 10µM stattic were detected by flow cytometry. Representative histograms of each group were shown. (e) The CTL activity of conventional TCR-T, IL-21R-TCR-T and IL-21R-TCR-T with 10µM stattic after 36h coculture with HepG2 was measured by LDH assay (n = 4). (f) The STAT3 phosphorylation level of conventional TCR-T and IL-21R-TCR-T after 30, 45 and 60 days in vitro culture was detected by flow cytometry. The representative histogram and the MFI of pSTAT3 (g) were shown (n = 3). The data was shown as Mean ± SEM, ****p <*0.001, *****p <*0.0001.


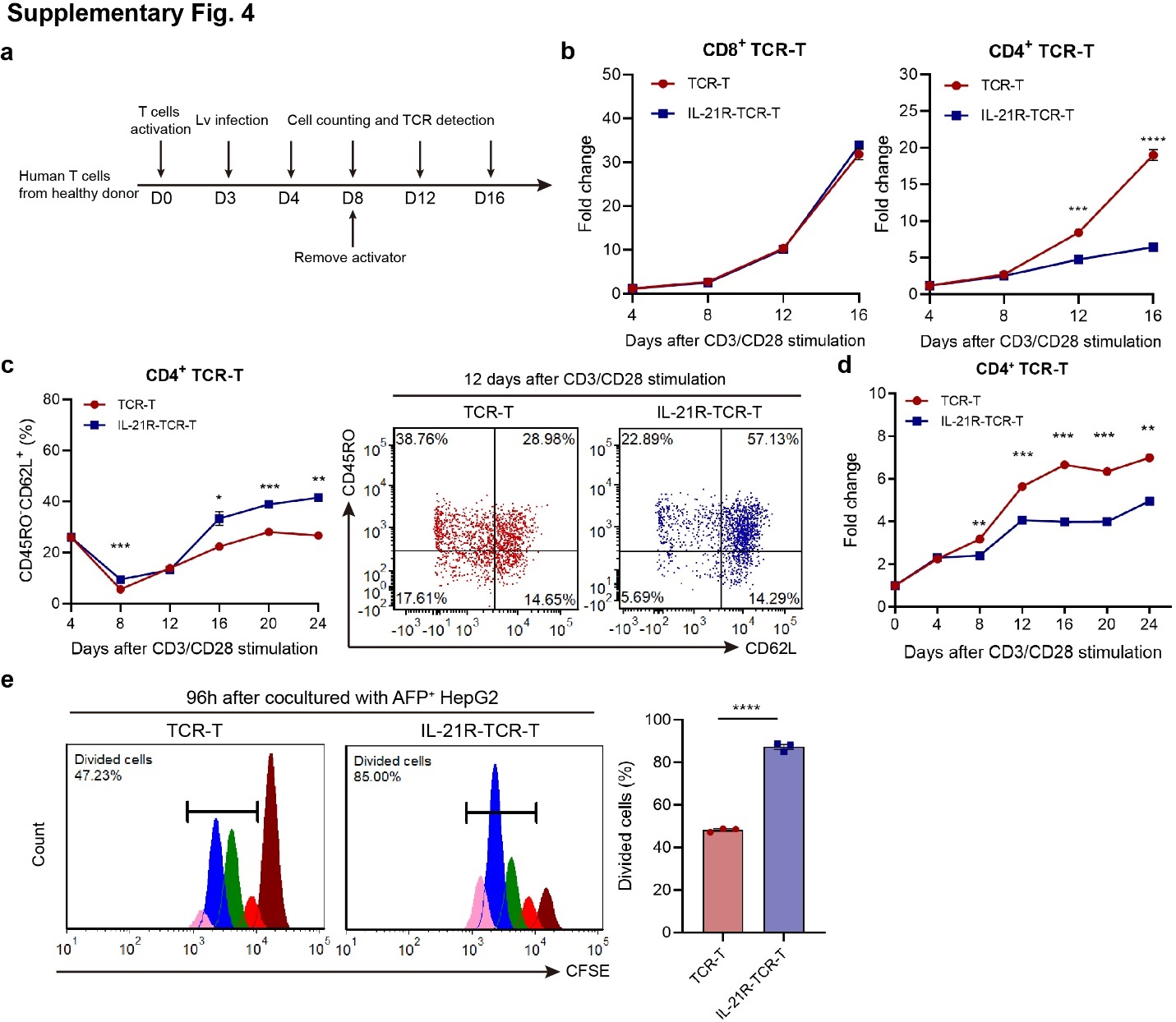


Figure. S4. Proliferation of IL-21R-TCR-T and memory phenotype of CD4^+^ TCR-T subsets. (a) The ex vivo generating process of TCR-T and IL-21R-TCR-T. (b) The proliferating fold change of CD8^+^ and CD4^+^ TCR-T and IL-21R-TCR-T during the initial generating process was shown respectively (n = 3). (c) The percentage of the CD45RO^-^CD62L^+^ population in CD4^+^ TCR-T or IL-21R-TCR-T from Day 4 to Day 24 after CD3/CD28 activation was monitored by flow cytometry (n = 3). The CD45RO and CD62L expression of CD4^+^ TCR-T and IL-21R-TCR-T 12 days after CD3/CD28 activation was shown. (d) The proliferating fold change of CD4^+^ TCR-T and IL-21R-TCR-T after CD3/CD28 activation was shown (n = 3). (e) CFSE labeled TCR-T or IL-21R-TCR-T were cocultured with HepG2 for 96h and analyzed by flow cytometry. The brown peak indicated the first proliferating generation, the red indicated the second, the green indicated the third, the blue indicated the fourth and the pink indicated the fifth. The percentage of divided cells in the TCR^+^ population was shown (n = 3). Data were shown as Mean ± SEM, **p <*0.05, ***p <*0.01, ****p <*0.001, *****p <*0.0001.


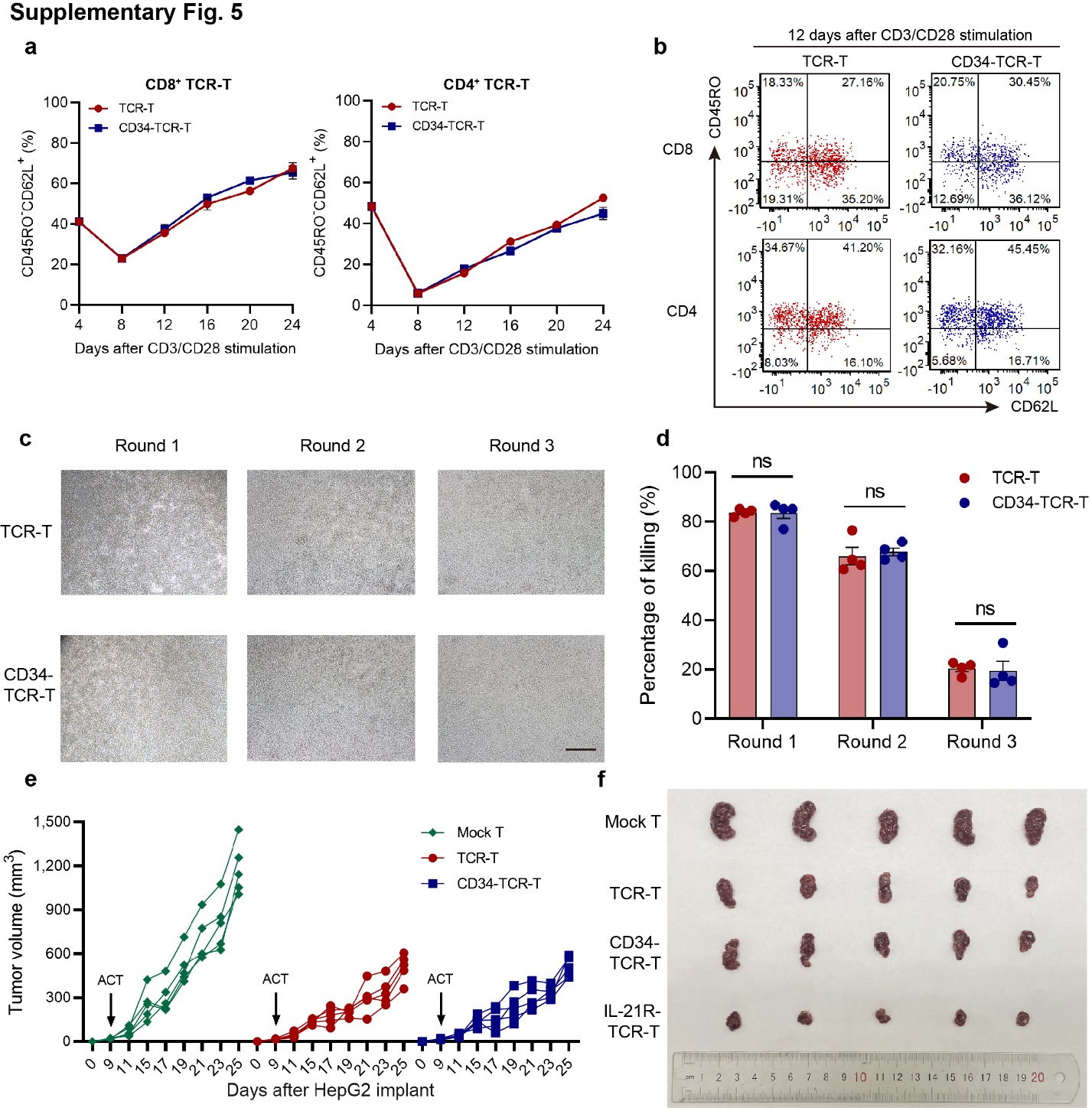


Figure. S5. The proliferation, memory phenotype and antitumor function of CD34-TCR-T. (a) The percentage of the CD45RO^-^CD62L^+^ population in CD8^+^ and CD4^+^ TCR-T or CD34-TCR-T from Day 4 to Day 24 after CD3/CD28 activation was monitored by flow cytometry (n = 3). (b) The CD45RO and CD62L expression of CD8^+^ and CD4^+^ TCR-T and CD34-TCR-T 12 days after CD3/CD28 activation was shown. (c) The CTL activity of conventional TCR-T and CD34-TCR-T after multiple-round coculture with HepG2 was measured by LDH assay (n = 4). Scale bar =100 μm. (d) Representative pictures of HepG2 after 36h coculture (Round 1, Round 2 and Round 3, the same procedure as Fig. 1a) with conventional TCR-T or CD34-TCR-T were shown. (e) The tumor volume of each mouse after HepG2 implantation and mock-transduced T, TCR-T or CD34-TCR-T transfer was monitored (n = 5). (f) Tumors from tumor-bearing mice receiving mock-transduced T, TCR-T, CD34-TCR-T or IL-21R-TCR-T treatment were isolated at the end of the experiment. The picture of isolated tumors was shown (n = 5). Data were shown as Mean ± SEM, NS, not significant.


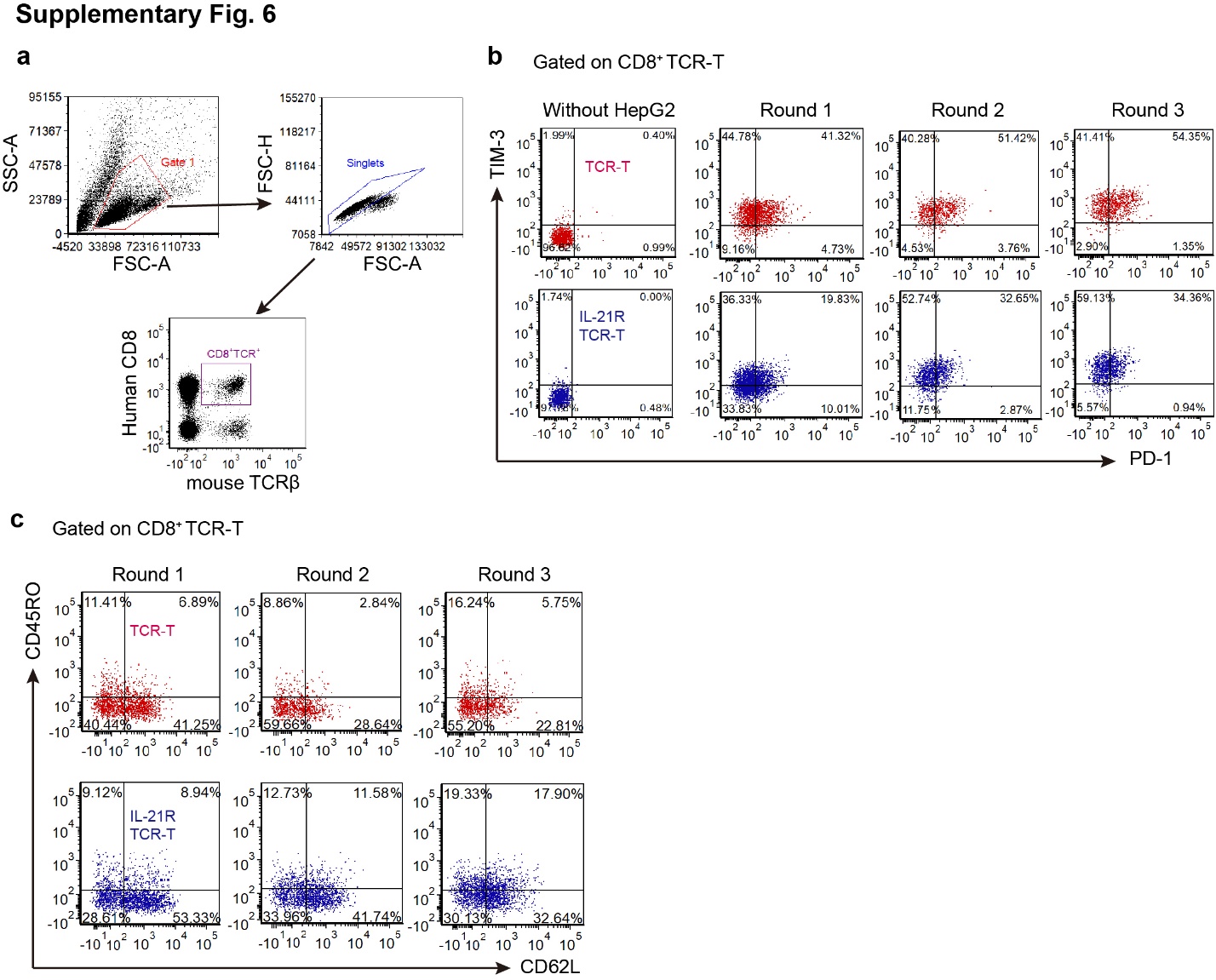


Figure. S6. Expression of exhaustion and memory related markers in CD8^+^ IL-21R-TCR-T during repetitive tumor stimulation. (a) The gating strategy of CD8^+^ TCR-T subsets in conventional TCR-T and IL-21R-TCR-T during repetitive coculture assay was shown. The expression of PD-1, TIM-3 (b) and CD45RO, CD62L (c) in CD8^+^ TCR-T subsets of conventional TCR-T and IL-21R-TCR-T after 36h coculture with HepG2 was detected by flow cytometry. Representative pictures of the dot plots at each timepoint were shown.


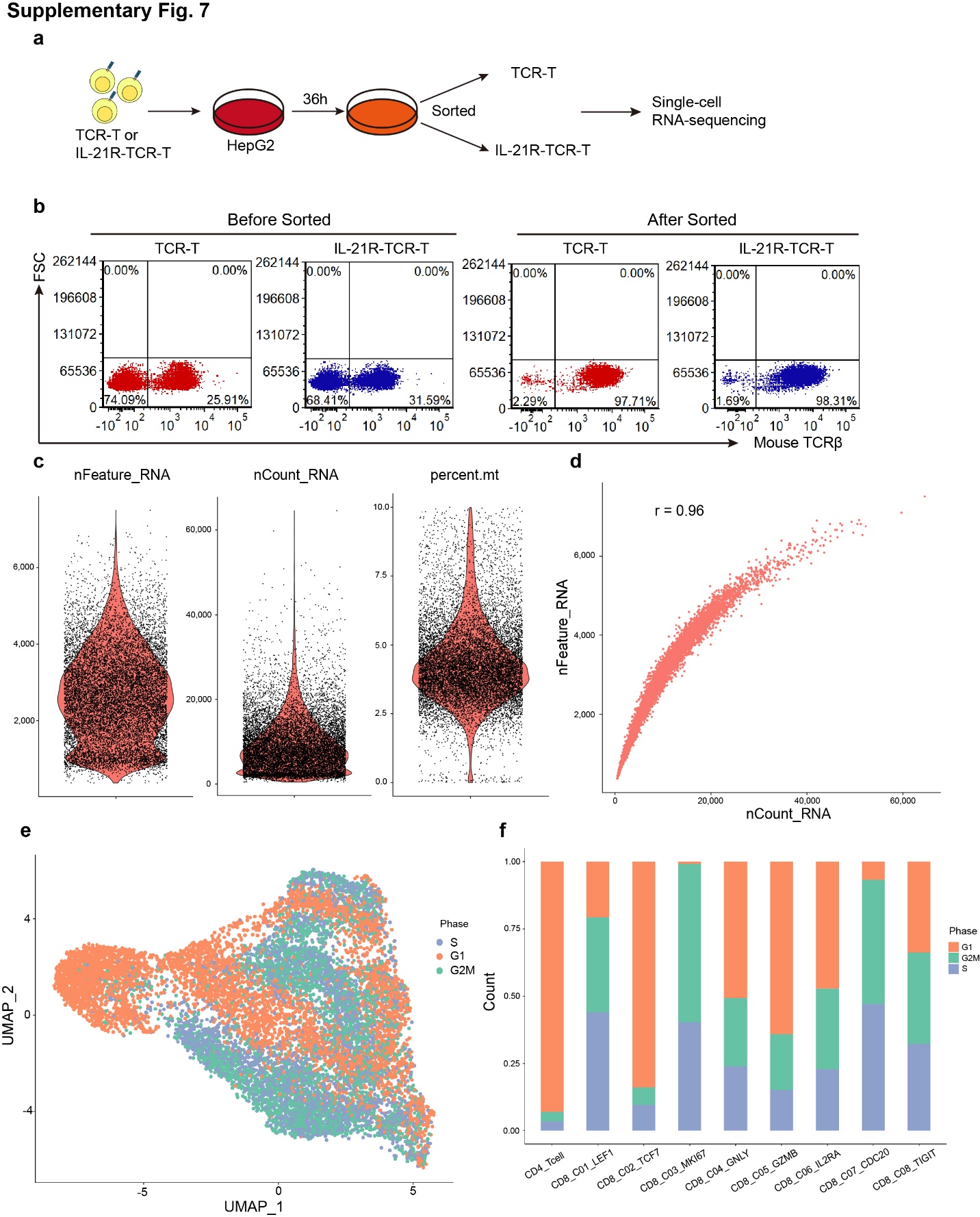


Figure. S7. Cells preparation procedure and basic information of the single-cell RNA-seq data. (a) IL-21R-TCR-T or TCR-T after 36h cocultured with HepG2 were sorted and analyzed by single-cell RNA sequencing. The procedure was shown. (b) The percentage of AFP-TCR^+^ proportion in TCR-T and IL-21R-TCR-T before and after sorting was measured by flow cytometry. (c) Violin plots showed the distribution of the number of detected genes per cell, the number of total UMI counts per cell, and the proportion of mitochondrial gene counts. (d) The scatter plot showed the correlation between the number of detected genes per cell and the number of total UMI counts per cell. (e) UMAP plot (Fig. 7a) colored by cell cycle phase. (f) Bar plot showing the contribution of the cell cycle phase to each cell type.


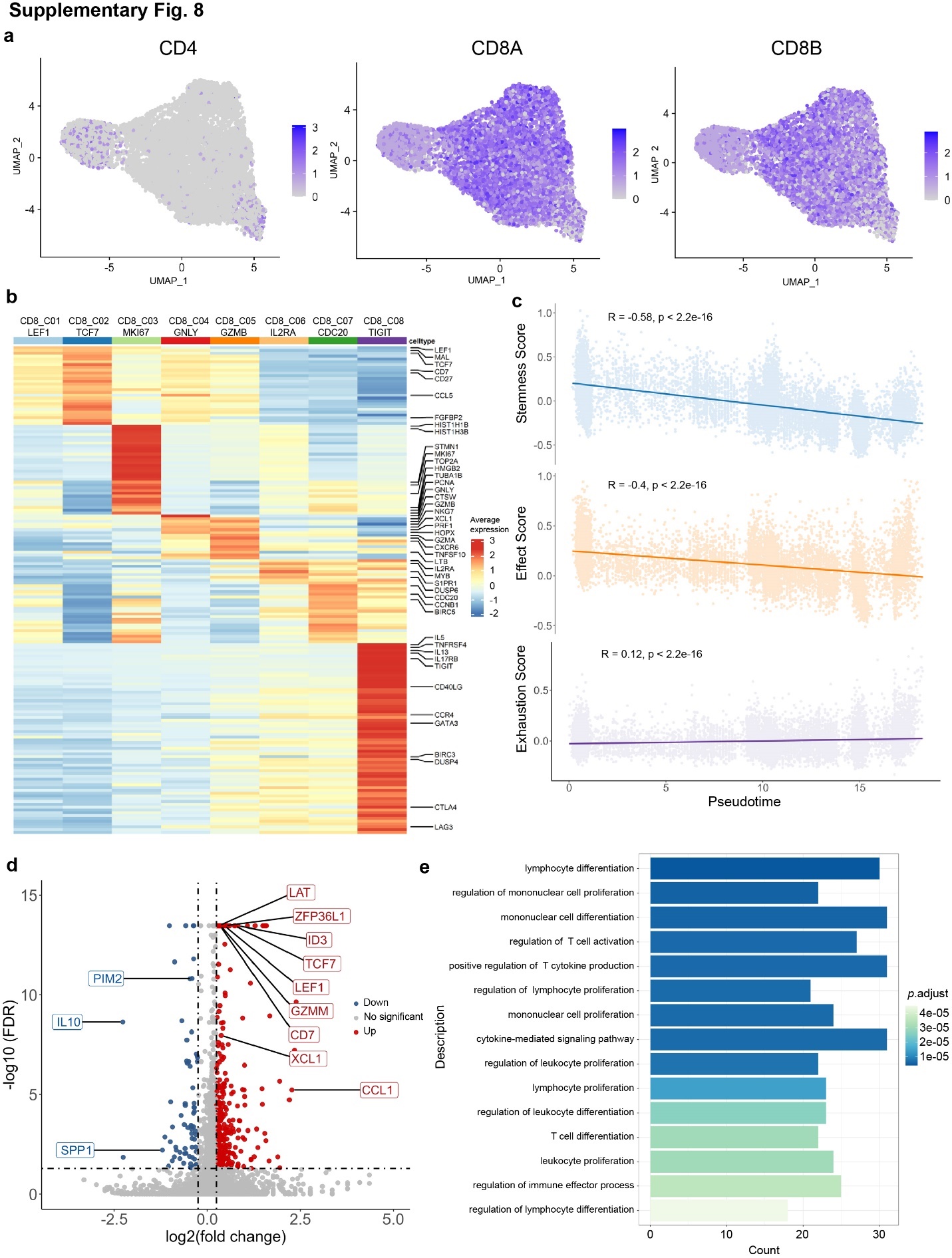


Figure. S8. Cluster Characterization of T cells and DEGs analysis between IL-21R-TCR-T and TCR-T. (a) The canonical marker genes expression to distinguish CD4^+^ T cells and CD8^+^ T cells. (b) Gene expression heatmap of 8 CD8^+^ T cell clusters. Rows represent signature genes and columns represent different clusters. The expression value is the z-score normalized mean expression. (c) Correlation of immune related score and pseudotime defined by monocle2 trajectory plot of all CD8^+^ T cells. Each dot represents a cell (Pearson’s correlation analysis). (d) Volcano plot showing differentially expressed genes between CD8^+^ TCR-T cells and CD8^+^ IL-21R-TCR-T cells. Red dots are upregulated significant genes (log2 (FC) > 0.25, FDR < 0.05), and blue dots are downregulated significant genes (log2 (FC) < -0.25, FDR < 0.05). (e) Enriched GO terms of differentially expressed genes in D.


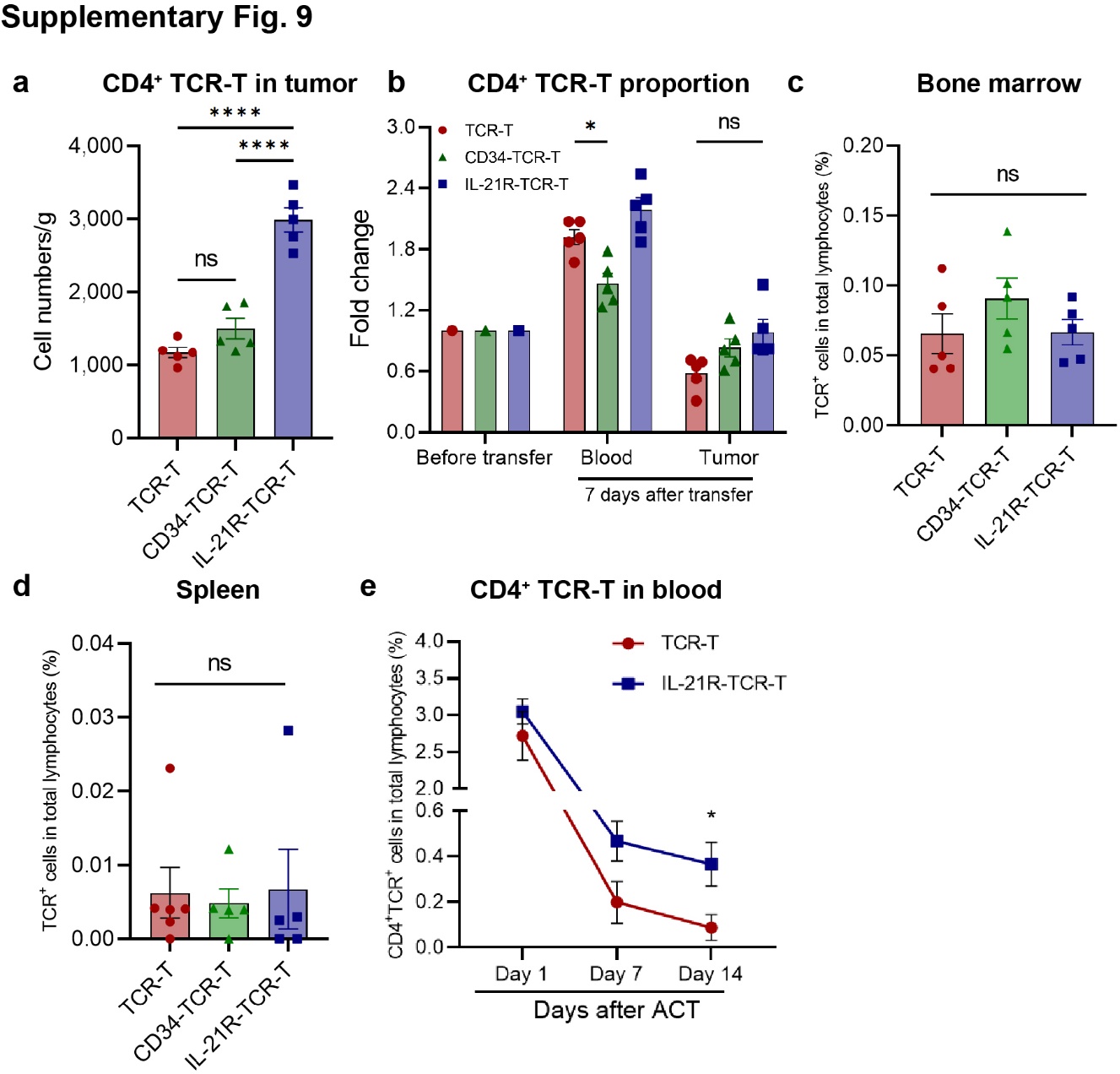


Figure. S9. CD4^+^ TCR-T in tumor and peripheral blood and monitoring of bone marrow and spleen cells after transfer. (a) The numbers of tumor-infiltrating CD4^+^ TCR-T in mice receiving TCR-T, CD34-TCR-T, and IL-21R-TCR-T were measured by flow cytometry and shown (n = 5). (b) The CD4^+^ TCR-T percentage in TCR-T, CD34-TCR-T and IL-21R-TCR-T before and after 7 days of transfer was measured by flow cytometry. The fold change of CD4^+^ TCR-T percentage in peripheral blood and tumor 7 days after transfer was shown (n = 5). The percentage of TCR^+^ cells in mice bone marrow (c) and spleen (d) 7 days after transfer was shown (n = 5). (e) The percentage of CD4^+^ TCR-T cells in total lymphocytes (mice and human lymphocytes) in the peripheral blood of each mouse 1, 7 and 14 days after TCR-T transfer was shown (n = 5). Data were shown as Mean ± SEM, **p <*0.05, ***p <*0.01, ****p <*0.001, *****p <*0.0001, NS, not significant.


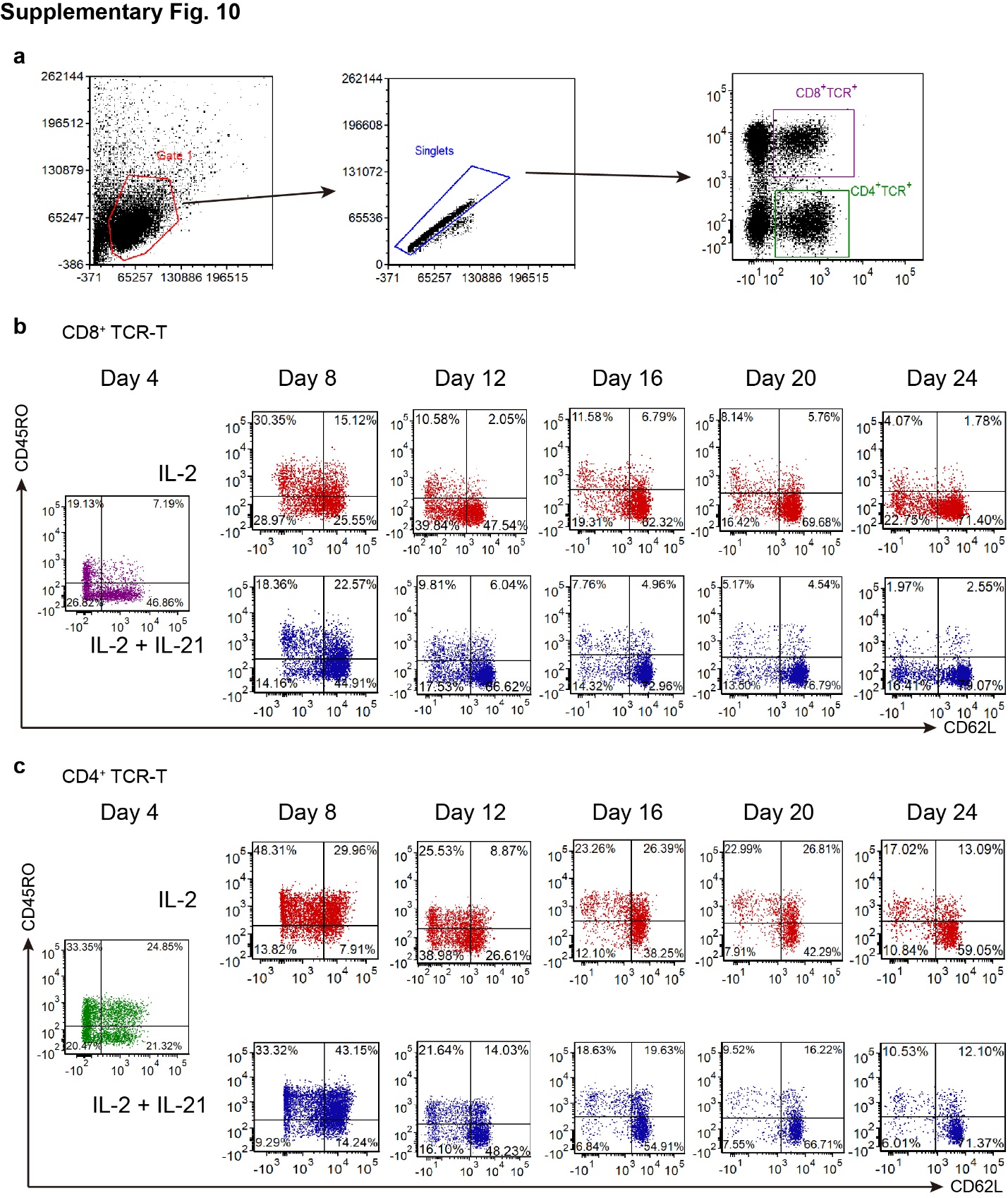


Figure. S10. Memory phenotype of the CD8^+^ and CD4^+^ TCR-T during in vitro culture at each time point. (a) The gating strategy of the CD8^+^ and CD4^+^ TCR-T subsets of TCR-T cultured in the presence of IL-2 or IL-2 and IL-21 was shown. The representative dot plots of CD45RO and CD62L expression at each timepoint in CD8^+^ TCR-T (b) and CD4^+^ TCR-T (c) from Day 4 to Day 24 were shown.


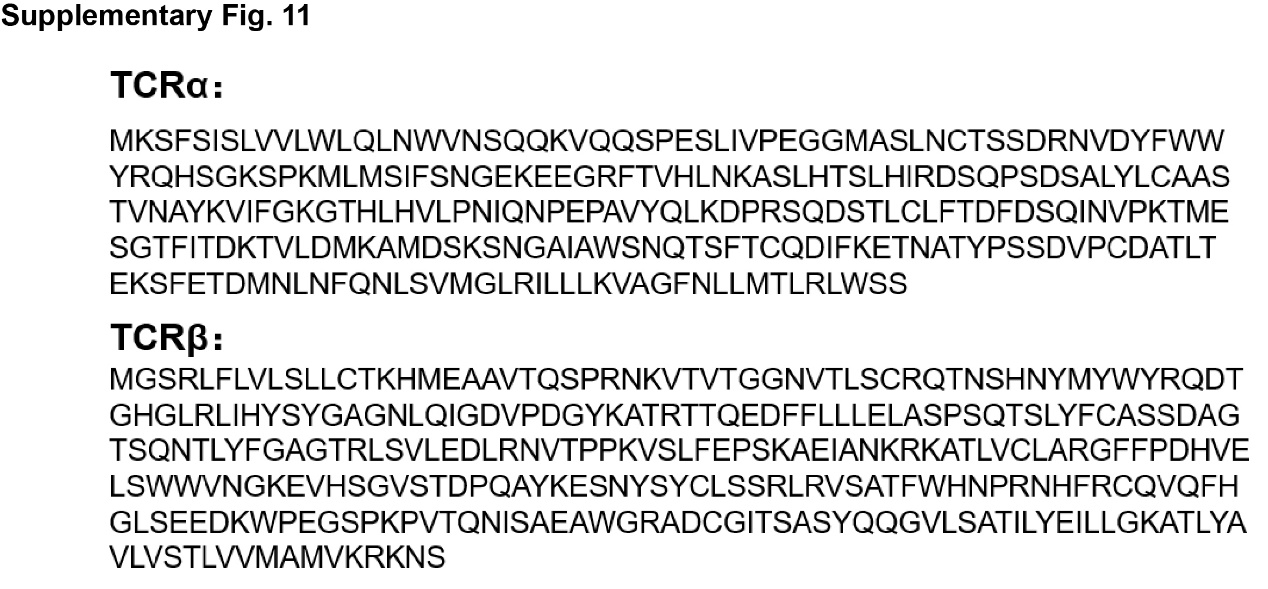


Figure. S11. The TCR α and β chain sequence of AFP-TCR-T.

Table S1 Overview of CD8^+^ T cell cluster characteristics.

Table S2 Gene markers for computing the immune-related score.

| Gene | Feature | Gene | Feature |
| --- | --- | --- | --- |
| TCF7 | stemness | CST7 | effect |
| SELL | stemness | ZWINT | proliferation |
| LEF1 | stemness | E2F1 | proliferation |
| CCR7 | stemness | FEN1 | proliferation |
| IL7R | stemness | FOXM1 | proliferation |
| FOXP1 | stemness | H2AFZ | proliferation |
| BACH2 | stemness | HMGB2 | proliferation |
| CD27 | stemness | MCM2 | proliferation |
| ID3 | stemness | MCM3 | proliferation |
| LTB | stemness | MCM4 | proliferation |
| CX3CR1 | effect | MCM5 | proliferation |
| IL2 | effect | MCM6 | proliferation |
| PRF1 | effect | MKI67 | proliferation |
| GZMA | effect | MYBL2 | proliferation |
| GZMB | effect | PCNA | proliferation |
| KLRG1 | effect | PLK1 | proliferation |
| NKG7 | effect | CCND1 | proliferation |
| IFNG | effect | AURKA | proliferation |
| FGFBP2 | effect | BUB1 | proliferation |
| EOMES | effect | TOP2A | proliferation |
| ID2 | effect | TYMS | proliferation |
| TBX21 | effect | DEK | proliferation |
| CD28 | effect | CCNB1 | proliferation |
| GZMH | effect | CCNE1 | proliferation |
| CD7 | effect | LAYN | exhaustion |
| GNLY | effect | ITGAE | exhaustion |
| FCGR3A | effect | PDCD1 | exhaustion |
| GZMK | effect | CTLA4 | exhaustion |
| LYAR | effect | HAVCR2 | exhaustion |
| GZMM | effect | LAG3 | exhaustion |
| TXNIP | effect | TIGIT | exhaustion |
| FCRL6 | effect | TOX | exhaustion |
| KLRD1 | effect | ENTPD1 | exhaustion |
|  |  | PRDM1 | exhaustion |

Table S3 Jaccard similarity between proliferating T-cell clusters and other clusters.

|  | CD8_C03_MKI67 | CD8_C07_CDC20 |
| --- | --- | --- |
| CD8_C01_LEF1 | 0.071428571 | 0.035375324 |
| CD8_C02_TCF7 | 0.024765158 | 0 |
| CD8_C04_GNLY | 0.020408163 | 0.001669449 |
| CD8_C05_GZMB | 0.011804384 | 0.005025126 |
| CD8_C06_IL2RA | 0.01095198 | 0.133144476 |
| CD8_C08_TIGIT | 0.003344482 | 0.057268722 |

Table S4 Regulon specificity score (RSS) for regulons in TCR-T cells and IL-21R-TCR-T cells.

|  | TCRT | IL21R_TCRT | TCRT_rank | IL21R_TCRT_rank |
| --- | --- | --- | --- | --- |
| JUNB_extended (70g) | 0.668305353 | 0.217151491 | 1 | 24 |
| YY1 (1418g) | 0.666451169 | 0.22257948 | 2 | 4 |
| JUND_extended (259g) | 0.666171498 | 0.222553683 | 3 | 5 |
| JUND (81g) | 0.665941635 | 0.221453472 | 4 | 10 |
| GTF2A2 (11g) | 0.665239579 | 0.220820623 | 5 | 13 |
| YBX1_extended (1808g) | 0.664617397 | 0.221369089 | 6 | 11 |
| FOS (1903g) | 0.66440264 | 0.22180037 | 7 | 8 |
| JUNB (20g) | 0.661212079 | 0.215611593 | 8 | 29 |
| JUN_extended (373g) | 0.656178775 | 0.221716745 | 9 | 9 |
| JUN (148g) | 0.649073057 | 0.220878691 | 10 | 12 |
| POLE4_extended (1102g) | 0.646950179 | 0.219728721 | 11 | 18 |
| KLF6_extended (28g) | 0.645844098 | 0.219962072 | 12 | 17 |
| STAT1_extended (740g) | 0.642130994 | 0.22336076 | 13 | 3 |
| IKZF1_extended (693g) | 0.637753603 | 0.220148071 | 14 | 15 |
| STAT3_extended (951g) | 0.632669541 | 0.22378403 | 15 | 2 |
| SF1 (297g) | 0.629857287 | 0.218337732 | 16 | 22 |
| POLE3_extended (797g) | 0.626941445 | 0.220452395 | 17 | 14 |
| RUNX3_extended (154g) | 0.626905311 | 0.220100698 | 18 | 16 |
| STAT1 (323g) | 0.624079559 | 0.222138216 | 19 | 6 |
| MYB_extended (172g) | 0.622123307 | 0.199276714 | 20 | 49 |
| STAT3 (119g) | 0.620415745 | 0.216566248 | 21 | 26 |
| KDM5A (682g) | 0.619659853 | 0.215495198 | 22 | 30 |
| ZNF706 (17g) | 0.619191214 | 0.208744562 | 23 | 41 |
| RUNX3 (97g) | 0.619166793 | 0.216755038 | 24 | 25 |
| SAP30 (119g) | 0.618846349 | 0.210513594 | 25 | 36 |
| TFDP1_extended (541g) | 0.617920681 | 0.213681752 | 26 | 32 |
| ATF4 (68g) | 0.615850838 | 0.211407866 | 27 | 34 |
| SMARCC2 (1189g) | 0.608970563 | 0.216402205 | 28 | 27 |
| ATF4_extended (81g) | 0.60640527 | 0.209043257 | 29 | 39 |
| IRF1 (105g) | 0.603126367 | 0.211296963 | 30 | 35 |
| RBPJ (46g) | 0.58889487 | 0.193110862 | 31 | 55 |
| IKZF1 (49g) | 0.587969101 | 0.21919898 | 32 | 21 |
| MYBL2_extended (258g) | 0.584768355 | 0.208493916 | 33 | 42 |
| MYBL2 (257g) | 0.584705138 | 0.208493285 | 34 | 43 |
| NFKB1_extended (44g) | 0.582139257 | 0.219655083 | 35 | 19 |
| E2F4 (171g) | 0.582098454 | 0.209843963 | 36 | 37 |
| ZBTB7A_extended (309g) | 0.579120403 | 0.209800824 | 37 | 38 |
| BATF_extended (64g) | 0.57880101 | 0.215893184 | 38 | 28 |
| KLF13_extended (281g) | 0.577698074 | 0.219650069 | 39 | 20 |
| TBX21_extended (200g) | 0.563097781 | 0.1954182 | 40 | 52 |
| MYB (79g) | 0.560812392 | 0.182505599 | 41 | 69 |
| UBTF (377g) | 0.560697136 | 0.214915252 | 42 | 31 |
| TBX21 (152g) | 0.558614062 | 0.194484627 | 43 | 53 |
| LEF1_extended (78g) | 0.54971977 | 0.225675072 | 44 | 1 |
| IRF2_extended (432g) | 0.53919719 | 0.212816338 | 45 | 33 |
| TFDP1 (135g) | 0.53109228 | 0.199550178 | 46 | 48 |
| E2F1_extended (1068g) | 0.530513166 | 0.202887146 | 47 | 45 |
| FOXP1_extended (463g) | 0.530460061 | 0.22206075 | 48 | 7 |
| E2F8 (154g) | 0.508454328 | 0.190474566 | 49 | 56 |
| FOXP1 (243g) | 0.502056759 | 0.218066825 | 50 | 23 |
| E2F8_extended (188g) | 0.501052723 | 0.188090748 | 51 | 61 |
| IRF2 (176g) | 0.499808054 | 0.205974334 | 52 | 44 |
| E2F1 (637g) | 0.499338132 | 0.200762516 | 53 | 47 |
| SP4_extended (829g) | 0.498512487 | 0.184658427 | 54 | 66 |
| PRDM1_extended (61g) | 0.490390176 | 0.160256077 | 55 | 95 |
| BPTF (15g) | 0.485836658 | 0.200996025 | 56 | 46 |
| NFYB_extended (837g) | 0.48133672 | 0.180577468 | 57 | 73 |
| NR3C1_extended (1526g) | 0.480443773 | 0.195442323 | 58 | 51 |
| E2F7 (379g) | 0.480317218 | 0.187077218 | 59 | 62 |
| NR3C1 (1348g) | 0.480208923 | 0.195857558 | 60 | 50 |
| NFYB (724g) | 0.474580939 | 0.178176991 | 61 | 75 |
| E2F7_extended (423g) | 0.471498234 | 0.183552971 | 62 | 68 |
| XBP1_extended (32g) | 0.469554634 | 0.208799971 | 63 | 40 |
| GATA3_extended (78g) | 0.461904207 | 0.173140259 | 64 | 83 |
| SMARCA4 (287g) | 0.449806032 | 0.189065043 | 65 | 59 |
| STAT5A_extended (704g) | 0.439602476 | 0.185580769 | 66 | 64 |
| BCL3_extended (30g) | 0.436847145 | 0.176665199 | 67 | 77 |
| SIN3A (1222g) | 0.434166305 | 0.186128014 | 68 | 63 |
| CHD2_extended (304g) | 0.434106839 | 0.190284012 | 69 | 57 |
| EP300 (548g) | 0.431306213 | 0.178217414 | 70 | 74 |
| SP1_extended (331g) | 0.431303982 | 0.174324042 | 71 | 80 |
| E2F2_extended (405g) | 0.429417202 | 0.174759582 | 72 | 79 |
| TAF1_extended (1516g) | 0.428249042 | 0.182481238 | 73 | 70 |
| E2F2 (309g) | 0.425099261 | 0.174318023 | 74 | 81 |
| CHD2 (216g) | 0.424081314 | 0.189709632 | 75 | 58 |
| SRF_extended (1173g) | 0.417505754 | 0.162195649 | 76 | 93 |
| NFATC3_extended (18g) | 0.412946377 | 0.180765797 | 77 | 72 |
| FOXO3_extended (111g) | 0.412846165 | 0.175314035 | 78 | 78 |
| NFATC3 (13g) | 0.410819656 | 0.181930778 | 79 | 71 |
| THAP11_extended (1107g) | 0.403485412 | 0.164166791 | 80 | 89 |
| ELF2_extended (1656g) | 0.400399296 | 0.165876264 | 81 | 87 |
| ELF2 (1205g) | 0.397093702 | 0.164635087 | 82 | 88 |
| ATF6_extended (755g) | 0.394163403 | 0.163893384 | 83 | 90 |
| ZMIZ1_extended (853g) | 0.392900388 | 0.159101364 | 84 | 96 |
| FOXP3_extended (20g) | 0.392157682 | 0.16278301 | 85 | 92 |
| RELB_extended (77g) | 0.390610713 | 0.184866177 | 86 | 65 |
| RELB (59g) | 0.387799995 | 0.184267055 | 87 | 67 |
| NFYC_extended (609g) | 0.386779892 | 0.1617173 | 88 | 94 |
| RAD21 (17g) | 0.384572747 | 0.155140062 | 89 | 100 |
| FOSB_extended (32g) | 0.381243534 | 0.148276333 | 90 | 106 |
| ELK4_extended (1507g) | 0.380591433 | 0.169939055 | 91 | 85 |
| FOSB (20g) | 0.379704054 | 0.148333126 | 92 | 105 |
| FOXO1_extended (939g) | 0.376671529 | 0.172820592 | 93 | 84 |
| REL_extended (128g) | 0.375735161 | 0.193577835 | 94 | 54 |
| FOXO1 (847g) | 0.374603257 | 0.173612566 | 95 | 82 |
| BATF (44g) | 0.366060724 | 0.17812451 | 96 | 76 |
| GTF2B_extended (252g) | 0.361357095 | 0.16378171 | 97 | 91 |
| REL (70g) | 0.35611283 | 0.188452387 | 98 | 60 |
| SREBF2_extended (1117g) | 0.35425431 | 0.168971386 | 99 | 86 |
| SP3_extended (1092g) | 0.353991215 | 0.158337075 | 100 | 97 |
| RCOR1_extended (666g) | 0.346593917 | 0.154107198 | 101 | 101 |
| TAF1 (954g) | 0.346108094 | 0.156589693 | 102 | 99 |
| NFYC (335g) | 0.334728833 | 0.149924544 | 103 | 104 |
| IRF3_extended (340g) | 0.321030573 | 0.147288824 | 104 | 107 |
| CEBPB_extended (589g) | 0.320409631 | 0.142625732 | 105 | 109 |
| PKNOX1_extended (109g) | 0.316740044 | 0.132609733 | 106 | 113 |
| ATF1_extended (606g) | 0.309435126 | 0.134478687 | 107 | 112 |
| ELK3_extended (394g) | 0.296545731 | 0.108779606 | 108 | 135 |
| ELK1_extended (553g) | 0.293901131 | 0.124002425 | 109 | 121 |
| ATF5_extended (649g) | 0.288486646 | 0.13204911 | 110 | 114 |
| GATA3 (34g) | 0.287791892 | 0.118593618 | 111 | 126 |
| THAP11 (385g) | 0.286365121 | 0.131605252 | 112 | 115 |
| TP53_extended (438g) | 0.286258378 | 0.128237251 | 113 | 117 |
| THAP1_extended (666g) | 0.284922066 | 0.128005221 | 114 | 118 |
| POLE3 (49g) | 0.280368326 | 0.14996281 | 115 | 102 |
| NR2C2_extended (586g) | 0.278987399 | 0.124749637 | 116 | 120 |
| GABPB1_extended (666g) | 0.277145446 | 0.124775754 | 117 | 119 |
| STAT5B_extended (416g) | 0.27559752 | 0.128378865 | 118 | 116 |
| HIVEP1_extended (133g) | 0.270108016 | 0.149960467 | 119 | 103 |
| RBBP5_extended (732g) | 0.267839041 | 0.121803121 | 120 | 122 |
| KLF9_extended (87g) | 0.267025014 | 0.157676395 | 121 | 98 |
| SREBF1_extended (453g) | 0.264285722 | 0.11345621 | 122 | 132 |
| STAT2_extended (236g) | 0.257176443 | 0.120699943 | 123 | 124 |
| GMEB1_extended (364g) | 0.252398857 | 0.116411638 | 124 | 129 |
| HIC1_extended (22g) | 0.246693506 | 0.118339431 | 125 | 127 |
| NFKB2_extended (99g) | 0.242421604 | 0.143775426 | 126 | 108 |
| ATF5 (329g) | 0.23525477 | 0.104541255 | 127 | 140 |
| SREBF2 (131g) | 0.23503132 | 0.141404407 | 128 | 110 |
| CREM_extended (12g) | 0.234326867 | 0.117316711 | 129 | 128 |
| KLF2_extended (68g) | 0.230015594 | 0.091979194 | 130 | 155 |
| NFKB2 (90g) | 0.230013839 | 0.139948472 | 131 | 111 |
| SRF (141g) | 0.229932129 | 0.106886562 | 132 | 137 |
| IRF7_extended (154g) | 0.228708095 | 0.120187551 | 133 | 125 |
| IRF3 (55g) | 0.225743771 | 0.11607598 | 134 | 130 |
| GABPA_extended (427g) | 0.225546513 | 0.107564734 | 135 | 136 |
| CHD1_extended (43g) | 0.223473378 | 0.098831921 | 136 | 146 |
| SP1 (192g) | 0.217678138 | 0.105922561 | 137 | 138 |
| RUNX2_extended (131g) | 0.213256781 | 0.114832692 | 138 | 131 |
| EGR2 (67g) | 0.207744138 | 0.109015326 | 139 | 134 |
| SP2_extended (279g) | 0.206283775 | 0.094171489 | 140 | 150 |
| IRF7 (108g) | 0.201968739 | 0.104125937 | 141 | 141 |
| FOXJ3_extended (128g) | 0.198740687 | 0.096664216 | 142 | 148 |
| E2F3_extended (231g) | 0.19809516 | 0.092517258 | 143 | 154 |
| USF2_extended (286g) | 0.196385394 | 0.093429392 | 144 | 152 |
| ELK1 (171g) | 0.195148557 | 0.090053122 | 145 | 159 |
| E2F3 (213g) | 0.193905025 | 0.091831403 | 146 | 156 |
| STAT2 (97g) | 0.192845385 | 0.092600685 | 147 | 153 |
| RUNX2 (123g) | 0.192351077 | 0.102879425 | 148 | 142 |
| FOXO3 (60g) | 0.191135111 | 0.101323035 | 149 | 144 |
| GABPA (312g) | 0.188790693 | 0.094420763 | 150 | 149 |
| GTF3C2 (224g) | 0.187976157 | 0.098336854 | 151 | 147 |
| ZNF384 (234g) | 0.187181 | 0.08975084 | 152 | 160 |
| ZNF143_extended (302g) | 0.186943027 | 0.085862751 | 153 | 164 |
| ZNF274_extended (287g) | 0.186797949 | 0.086392598 | 154 | 162 |
| MYC (159g) | 0.185993889 | 0.121334081 | 155 | 123 |
| GABPB1 (247g) | 0.185375413 | 0.090649549 | 156 | 158 |
| XRCC4_extended (259g) | 0.182891633 | 0.093731979 | 157 | 151 |
| TBL1XR1_extended (43g) | 0.18271763 | 0.112503478 | 158 | 133 |
| NFATC1_extended (89g) | 0.178396523 | 0.099976611 | 159 | 145 |
| ETV3_extended (239g) | 0.17780747 | 0.080697908 | 160 | 170 |
| BRF1_extended (286g) | 0.177328692 | 0.09142055 | 161 | 157 |
| HIVEP1 (57g) | 0.175835689 | 0.105052946 | 162 | 139 |
| CREBL2_extended (146g) | 0.174096426 | 0.08440859 | 163 | 167 |
| SP2 (198g) | 0.173683666 | 0.07569208 | 164 | 176 |
| KLF13 (11g) | 0.169624805 | 0.101776056 | 165 | 143 |
| FOXJ3 (59g) | 0.166148275 | 0.085792672 | 166 | 165 |
| TBP_extended (198g) | 0.164893975 | 0.076163281 | 167 | 174 |
| ESRRA_extended (264g) | 0.164471843 | 0.078939265 | 168 | 173 |
| NFIL3_extended (26g) | 0.164193234 | 0.083672537 | 169 | 168 |
| SETDB1_extended (249g) | 0.160707811 | 0.079147471 | 170 | 172 |
| TFE3_extended (152g) | 0.160497296 | 0.06997989 | 171 | 180 |
| BATF3_extended (21g) | 0.155881305 | 0.0736183 | 172 | 179 |
| ZNF76_extended (220g) | 0.153423097 | 0.065656117 | 173 | 186 |
| PHF8_extended (175g) | 0.151503934 | 0.069228922 | 174 | 182 |
| CREB1 (140g) | 0.149653235 | 0.068363223 | 175 | 183 |
| BDP1_extended (25g) | 0.148132757 | 0.088884651 | 176 | 161 |
| MAF_extended (50g) | 0.143013453 | 0.076027676 | 177 | 175 |
| ETV3 (140g) | 0.142301941 | 0.06588468 | 178 | 185 |
| ATF3_extended (90g) | 0.138028446 | 0.062978119 | 179 | 187 |
| HSF1_extended (98g) | 0.135048442 | 0.069961865 | 180 | 181 |
| RFX5_extended (112g) | 0.134895618 | 0.073658539 | 181 | 178 |
| NFE2L2_extended (141g) | 0.134480223 | 0.062284432 | 182 | 188 |
| NPDC1_extended (56g) | 0.13043566 | 0.080510059 | 183 | 171 |
| RXRA_extended (38g) | 0.125521025 | 0.073932215 | 184 | 177 |
| TFE3 (78g) | 0.124106916 | 0.048017842 | 185 | 199 |
| IRF4_extended (32g) | 0.124027496 | 0.085617166 | 186 | 166 |
| ZNF76 (154g) | 0.122145469 | 0.060631633 | 187 | 190 |
| MLXIP_extended (47g) | 0.12021444 | 0.083073013 | 188 | 169 |
| USF1_extended (99g) | 0.114479929 | 0.062077132 | 189 | 189 |
| EOMES_extended (49g) | 0.108063125 | 0.067525188 | 190 | 184 |
| RARG_extended (80g) | 0.106940047 | 0.059406333 | 191 | 191 |
| NFE2L3_extended (39g) | 0.106927522 | 0.086226399 | 192 | 163 |
| VDR_extended (17g) | 0.106699793 | 0.047952466 | 193 | 200 |
| FOSL2_extended (41g) | 0.104787829 | 0.046962385 | 194 | 202 |
| RFX5 (51g) | 0.100095959 | 0.055266761 | 195 | 195 |
| CEBPG_extended (76g) | 0.099275688 | 0.053400061 | 196 | 196 |
| RXRB_extended (98g) | 0.095472992 | 0.046722439 | 197 | 204 |
| EOMES (29g) | 0.092773512 | 0.052456924 | 198 | 197 |
| FOXN2_extended (70g) | 0.091954732 | 0.05005866 | 199 | 198 |
| MXI1 (49g) | 0.091186806 | 0.046855356 | 200 | 203 |
| HSF1 (61g) | 0.089971754 | 0.055624997 | 201 | 194 |
| CLOCK_extended (122g) | 0.089080307 | 0.047693794 | 202 | 201 |
| CREB3L2_extended (31g) | 0.085966441 | 0.028888542 | 203 | 230 |
| MAF (16g) | 0.084928049 | 0.044623946 | 204 | 206 |
| CHD1 (27g) | 0.083537679 | 0.041557399 | 205 | 208 |
| RXRA (18g) | 0.083412913 | 0.057142437 | 206 | 192 |
| AHRR (94g) | 0.079914542 | 0.037914131 | 207 | 213 |
| DBP_extended (45g) | 0.078410245 | 0.044391054 | 208 | 207 |
| ZNF143 (76g) | 0.077508432 | 0.036253523 | 209 | 217 |
| ZEB1_extended (21g) | 0.072850995 | 0.036471706 | 210 | 216 |
| KLF2 (15g) | 0.072781126 | 0.030635929 | 211 | 228 |
| HDAC2 (14g) | 0.071617179 | 0.057008231 | 212 | 193 |
| ETS2_extended (56g) | 0.071436389 | 0.031280747 | 213 | 226 |
| ERF_extended (92g) | 0.070614746 | 0.045777706 | 214 | 205 |
| RXRB (73g) | 0.070391357 | 0.036593756 | 215 | 215 |
| RARG (38g) | 0.069459088 | 0.041437674 | 216 | 210 |
| CEBPB (45g) | 0.069234403 | 0.036988213 | 217 | 214 |
| KLF16_extended (78g) | 0.067810238 | 0.039901257 | 218 | 212 |
| MTF1 (40g) | 0.061480352 | 0.02862576 | 219 | 231 |
| MAX (12g) | 0.060452022 | 0.034472071 | 220 | 219 |
| POLR3G_extended (72g) | 0.058541531 | 0.03303069 | 221 | 223 |
| USF1 (32g) | 0.055475415 | 0.031777219 | 222 | 225 |
| SETDB1 (55g) | 0.055354451 | 0.033460175 | 223 | 222 |
| CDK2AP1 (12g) | 0.054401531 | 0.034878004 | 224 | 218 |
| FOSL2 (13g) | 0.052812149 | 0.022413177 | 225 | 238 |
| PPARA_extended (44g) | 0.051518713 | 0.041454002 | 226 | 209 |
| PBX3_extended (50g) | 0.051435633 | 0.02696204 | 227 | 232 |
| IRF9_extended (25g) | 0.050137375 | 0.030814422 | 228 | 227 |
| VDR (12g) | 0.050089822 | 0.025425744 | 229 | 235 |
| ZNF770 (16g) | 0.048468777 | 0.032230167 | 230 | 224 |
| MBD4_extended (15g) | 0.046133672 | 0.030100266 | 231 | 229 |
| ATF7_extended (50g) | 0.045903651 | 0.023354393 | 232 | 236 |
| BACH1_extended (21g) | 0.045872692 | 0.026499917 | 233 | 233 |
| MLXIP (18g) | 0.043782036 | 0.040089133 | 234 | 211 |
| ARNT_extended (25g) | 0.042297312 | 0.020875344 | 235 | 241 |
| TGIF2 (25g) | 0.040661926 | 0.020445435 | 236 | 243 |
| CREB3L4_extended (42g) | 0.040604642 | 0.014887805 | 237 | 262 |
| E2F6_extended (49g) | 0.040232134 | 0.020524349 | 238 | 242 |
| HES6_extended (41g) | 0.038788845 | 0.017887146 | 239 | 253 |
| CD59_extended (15g) | 0.03821548 | 0.014696578 | 240 | 263 |
| FOXK1_extended (24g) | 0.036888976 | 0.019079949 | 241 | 248 |
| TCF3_extended (22g) | 0.036416742 | 0.018682352 | 242 | 251 |
| ZFY_extended (18g) | 0.0362191 | 0.026413619 | 243 | 234 |
| PBX3 (28g) | 0.036043531 | 0.018950232 | 244 | 249 |
| ETV2_extended (26g) | 0.034856748 | 0.016729577 | 245 | 257 |
| KLF7_extended (11g) | 0.034071672 | 0.034378376 | 246 | 220 |
| ETV2 (23g) | 0.034036297 | 0.015844302 | 247 | 260 |
| ZNF639 (22g) | 0.033928455 | 0.019634322 | 248 | 245 |
| NFYA_extended (17g) | 0.033493965 | 0.021903433 | 249 | 239 |
| NR2F6_extended (27g) | 0.03309947 | 0.033528297 | 250 | 221 |
| ZNF181 (12g) | 0.032554456 | 0.013889531 | 251 | 268 |
| FOXJ2_extended (20g) | 0.031470291 | 0.015931703 | 252 | 259 |
| CREB3_extended (26g) | 0.031177957 | 0.021511469 | 253 | 240 |
| RUNX1_extended (13g) | 0.027980807 | 0.013062833 | 254 | 270 |
| KLF12_extended (25g) | 0.027475181 | 0.023035261 | 255 | 237 |
| ATF7 (29g) | 0.027351578 | 0.01209214 | 256 | 274 |
| SNAI3_extended (15g) | 0.027178325 | 0.014260274 | 257 | 266 |
| BACH2_extended (23g) | 0.025663891 | 0.018568687 | 258 | 252 |
| RFXAP_extended (35g) | 0.025254993 | 0.019222514 | 259 | 247 |
| ZNF846 (22g) | 0.024926684 | 0.008255694 | 260 | 282 |
| MIOS_extended (18g) | 0.02492099 | 0.012824109 | 261 | 272 |
| HIVEP2_extended (14g) | 0.024061897 | 0.017176402 | 262 | 255 |
| RFXAP (28g) | 0.022832569 | 0.018692667 | 263 | 250 |
| TCF12_extended (12g) | 0.022624522 | 0.00975889 | 264 | 280 |
| TFAP4_extended (22g) | 0.02237944 | 0.020088179 | 265 | 244 |
| NRF1_extended (30g) | 0.022124647 | 0.014529251 | 266 | 265 |
| TFEB_extended (13g) | 0.022089723 | 0.011104931 | 267 | 276 |
| TFAP4 (19g) | 0.021868007 | 0.019406514 | 268 | 246 |
| SOX12 (14g) | 0.021850605 | 0.012520048 | 269 | 273 |
| NR2F6 (23g) | 0.021723494 | 0.016216764 | 270 | 258 |
| ZNF460_extended (15g) | 0.021306821 | 0.017490218 | 271 | 254 |
| MNT_extended (22g) | 0.021202739 | 0.01405707 | 272 | 267 |
| RARA_extended (14g) | 0.020792275 | 0.016880166 | 273 | 256 |
| FOXK2_extended (18g) | 0.020527366 | 0.015456647 | 274 | 261 |
| NR2C1_extended (15g) | 0.019888356 | 0.013544531 | 275 | 269 |
| BRF2_extended (15g) | 0.019659785 | 0.008420045 | 276 | 281 |
| RFX2_extended (23g) | 0.019653776 | 0.014682037 | 277 | 264 |
| NRF1 (25g) | 0.018996665 | 0.01177792 | 278 | 275 |
| MNT (18g) | 0.018666733 | 0.012927504 | 279 | 271 |
| THAP1 (18g) | 0.018560269 | 0.006087355 | 280 | 287 |
| RFX7_extended (11g) | 0.017886931 | 0.011018654 | 281 | 277 |
| CUX1_extended (11g) | 0.017360665 | 0.009941161 | 282 | 279 |
| E4F1 (13g) | 0.01533026 | 0.006828724 | 283 | 284 |
| HES6 (13g) | 0.015116515 | 0.002407963 | 284 | 292 |
| ERF (22g) | 0.014464092 | 0.005723349 | 285 | 289 |
| ESRRA (12g) | 0.014376525 | 0.010485462 | 286 | 278 |
| RARA (11g) | 0.013664241 | 0.008059731 | 287 | 283 |
| MAFK_extended (16g) | 0.013475817 | 0.006085774 | 288 | 288 |
| ADNP2 (13g) | 0.011604703 | 0.003573584 | 289 | 291 |
| POU2F1_extended (13g) | 0.011009098 | 0.006163641 | 290 | 286 |
| ZNF611 (12g) | 0.009828469 | 0.003900739 | 291 | 290 |
| E2F6 (12g) | 0.008300526 | 0.00648579 | 292 | 285 |
